# One-step knock-in CAR constructs in human NK cells enable scalable, TGFβ1-resistant immunotherapy for solid tumors

**DOI:** 10.7150/thno.127565

**Published:** 2026-03-25

**Authors:** Su-Min Yee, Ji Hye Jeong, Daeun Kim, Sung-Bae Kang, Hanna Yoon, Hyein Cho, Daechan Park, Eunsung Jun, Mihue Jang

**Affiliations:** 1 Medicinal Materials Research Center, Biomedical Research Division, Korea Institute of Science and Technology, Seoul 02792, Republic of Korea.; 2 Department of Convergence Medicine, Asan Institute for Life Sciences, University of Ulsan College of Medicine and Asan Medical Center, Seoul 05505, Republic of Korea.; 3 Institute of Chemical Processes, Seoul National University, Seoul 08826, Republic of Korea.; 4 Department of Molecular Science and Technology, Ajou University, Suwon 16499, Republic of Korea.; 5 Ajou Energy Science Research Center, Ajou University, Suwon 16499, Republic of Korea.; 6 KHU-KIST Department of Converging Science and Technology, Kyung Hee University, Seoul 02447, Republic of Korea.; 7 Rare & Pediatric Cancer Branch, Research Institute, National Cancer Center, Goyang 10408, Republic of Korea.; 8 College of Advanced Bio-Convergence Engineering, Ajou University, Suwon 16499, Republic of Korea.; 9 Division of Hepato-Biliary and Pancreatic Surgery, Department of Surgery, University of Ulsan College of Medicine, Asan Medical Center, Seoul 05505, Republic of Korea.; 10 Division of Bio-Medical Science and Technology, KIST School, University of Science and Technology (UST), Seoul 02792, Republic of Korea.

**Keywords:** CAR-NK, TGFBR2 knockout, homology-directed repair, glucocorticoid response element (GRE), pancreatic ductal adenocarcinoma

## Abstract

**Rationale:**

Chimeric antigen receptor (CAR)-engineered natural killer (NK) cells represent a promising modality for cancer immunotherapy, yet their efficacy in solid tumors is limited by immunosuppressive cues from the tumor microenvironment (TME), particularly, transforming growth factor β (TGFβ).

**Methods:**

Primary human NK cells were cytokine-activated (IL-12/15/18) and engineered via a one-step electroporation that delivered Cas9 ribonucleoprotein and a dsDNA donor (1 kb homology arms, SFFV promoter, poly(A), GRE element) to knock out TGFBR2 and knock in a mesothelin CAR. We compared against a two-step AAV method, and treated dexamethasone (Dex) during manufacture. Anti-tumor function was assessed using AsPC-1 cancer killing assays, patient-derived pancreatic cancer organoids (caspase-3/7 imaging, luciferase viability, live/dead FACS), and multi-omics profiling (RNA-seq, ATAC-seq, GSEA) to evaluate metabolic and transcriptional modifications.

**Results:**

We report a streamlined, one-step strategy that simultaneously disrupts the TGFβ receptor II (TGFβRII) and integrates a mesothelin-targeting CAR transgene into primary NK cells via electroporation. By optimizing single-guide RNAs, donor DNA templates incorporating glucocorticoid response elements, and electroporation parameters, we achieved markedly improved knock-in efficiency. Transient Dex treatment during genome editing enhanced CAR expression and cytotoxic function in both electroporation- and AAV-mediated platforms. Dex augmented the cytotoxic activity of CAR-NK cells by promoting oxidative phosphorylation and ATP production.

**Conclusions:**

We designed robust, TGFβ1-resistant allogeneic CAR-NK cells using a virus-free, one-step engineering strategy, establishing a versatile, clinically scalable method for engineering metabolically fortified CAR-NK cells capable of overcoming TME-mediated suppression in solid tumors.

## Introduction

Pancreatic ductal adenocarcinoma (PDAC) is a highly aggressive malignant tumor with poor prognosis, with a five-year survival rate of only approximately 13%, largely due to late diagnosis, limited therapeutic options, and resistance to existing treatments 1-4. One of the hallmarks of PDAC is its desmoplastic tumor microenvironment (TME), marked by a collagen-rich dense extracellular matrix (ECM) and a complex cellular network comprising cancer-associated fibroblasts (CAFs), immune cells, and other stromal components. This unique TME fosters immune evasion and restricts immune cell infiltration 5, 6. Among them, CAFs play a pivotal role in tumor progression and immunosuppression by secreting inhibitory factors, notably transforming growth factor β (TGFβ). TGFβ shapes the TME and modulates immune responses, contributing to the immunosuppressive milieu 7, 8.

Natural killer (NK) cells are a key component of the innate immune system and are attractive candidates for cancer immunotherapy because they can recognize and eradicate malignant or stressed cells without prior antigen sensitization 9, 10. NK-based adoptive therapies, including chimeric antigen receptor (CAR)-engineered NK cells, offer distinct advantages, including a reduced risk of cytokine release syndrome and allogeneic production feasibility 11. However, the immunosuppressive TME of PDAC remains a major barrier to NK cell efficacy. One of the most potent suppressors in the TME is TGFβ, which suppresses NK cell function through various mechanisms 12. TGFβ signaling downregulates NK activating receptors, such as NKG2D, NKp30, and DNAM-1, impairs metabolic fitness by reducing mTOR pathway activity and mitochondrial function, and inhibits proliferation and IFN-γ production 13-16. Moreover, TGFβ skews NK cells toward an ILC-1-like non-cytotoxic phenotype, further contributing to immune evasion and tumor progression 17.

In pancreatic cancer, CAFs are a major source of TGFβ, contributing not only to immunosuppression but also to ECM remodeling, fibrosis, and enhanced metastatic potential 18. The resulting dense stroma and altered chemokine gradients further hinder NK cell trafficking and persistence in the TME. To overcome TGFβ-mediated inhibition, various engineering strategies have been developed to render NK cells resistant to TGFβ signaling. These include the disruption of TGFβ receptor signaling through knockout or the use of dominant-negative forms of TGFβRII 19, 20, as well as the expression of decoy receptors or TGFβ trap molecules that sequester TGFβ within the TME 21. Additionally, CRISPR-mediated gene editing of key components in the TGFβ signaling pathway has been employed to enhance NK cell cytotoxicity and persistence 22. Another innovative approach involves the design of synthetic receptors, such as TGFβ-switch CAR constructs, which convert inhibitory TGFβ signals into activating stimuli to promote NK cell function 23. These approaches have demonstrated improved NK cell fitness, cytokine production, and anti-tumor activity in preclinical models, highlighting the therapeutic potential of engineering TGFβ-resistant NK cells for solid tumor immunotherapy.

Given the central role of TGFβ in immune evasion and NK cell dysfunction in PDAC, targeting this pathway is a promising strategy to improve the efficacy of CAR-NK cell therapies and overcome the barriers imposed by the hostile TME. The canonical TGFβ signaling cascade is initiated by the binding of TGFβ to its receptor complex (TGFβRI/TGFβRII), leading to the phosphorylation of SMAD2/3. The phosphorylated SMADs form a complex with SMAD4 and are translocated into the nucleus, where they regulate the expression of target genes 24. Various strategies have been developed to inhibit this pathway and restore NK cell function, including direct targeting of TGFβ signaling and genetic modification of NK cells to express dominant-negative TGFβRII 25, 26. However, the application of these approaches is often limited by technical challenges in gene engineering and the complexity of multi-step manufacturing protocols.

To overcome these challenges, we aimed to develop streamlined, one-step process for generating CAR-NK cells resistant to TGFβ-mediated suppression to enable simultaneous disruption of the *TGFβR2* gene and integration of the *CAR* transgene into human primary NK (pNK) cells. By blocking TGFβ signaling while endowing NK cells with tumor-targeting capabilities, this strategy aims to preserve cytotoxic function and overcome hurdles related to genetic manipulation and scalability. Through this approach, we seek to establish a robust and clinically relevant CAR-NK cell therapy capable of counteracting TGFβ-driven immune evasion in PDAC.

## Materials and Methods

### Cell culture

AsPC-1 cells were purchased from the Korea Cell Line Bank (KCLB; Seoul, South Korea), whereas K562 and HEK293T cells were acquired from the American Type Culture Collection (ATCC; VA, USA). AsPC-1 and K562 cells were cultured in RPMI 1640 medium (LM011-51, Welgene, Gyeongsan, South Korea) supplemented with 10% fetal bovine serum (FBS; S001-01, Welgene) and 1% antibiotic-antimycotic solution (AA; LS203-01, Welgene). HEK293T cells were cultured in Dulbecco's Modified Eagle Medium (DMEM; LM001-05, Welgene) supplemented with 10% FBS (Welgene), 1% AA (Welgene), and 10 mM HEPES (15630056, Gibco, Thermo Fisher Scientific, MA, USA). Cells were regularly tested with the mycoplasma detection kit (25237, Intronbio, WA, USA) and confirmed to be free from mycoplasma.

### Isolation and *ex vivo* expansion of human primary NK (pNK) cells

pNK cells were expanded from human peripheral blood mononuclear cells (PBMCs; CC-2705; Lonza, Morristown, NJ, USA) using mitomycin C-treated K562 feeder cells as previously described 16. Briefly, PBMCs were co-cultured with irradiated K562 feeder cells in NK MACS medium (130-114-429, Miltenyi Biotec, Bergisch Gladbach, Germany) supplemented with IL-2 (200 IU/mL; 202-IL-500, R&D Systems, MN, USA) and IL-15 (10 ng/mL; 200-15-250UG, PeproTech, NJ, USA) for 2-3 weeks. After expansion, NK cells were purified using a human NK cell isolation kit (Miltenyi Biotec) according to the manufacturer's instructions.

### Generation of genetically engineered pNK cells

To construct the donor double-stranded DNA (dsDNA) for CAR expression, DNA fragments encoding a second-generation CAR containing the SS1 single-chain variable fragment (scFv) (US Patent: US7081518) were synthesized by Cosmo Genetech (Seoul, Korea). Homology arms flanking exon 4 of the human *TGFBR2* gene were cloned and incorporated into the pCMV3 vector using the NEBuilder HiFi DNA assembly kit (E2621L, New England Biolabs, NEB; MA, USA). The assembled plasmid was used as a PCR template for donor DNA amplification, employing Phusion High-Fidelity DNA Polymerases (F-530L, Thermo Fisher Scientific). For genetic engineering, pNK cells were activated for 7 h using a cytokine cocktail consisting of IL-12 (10 ng/mL; 200-12H-50UG, PeproTech), IL-15 (50 ng/mL; PeproTech), and IL-18 (50 ng/mL; 592106, BioLegend, San Diego, CA, USA). For knock-in (KI) experiments, we used PCR-amplified linear dsDNA as the homology-directed repair (HDR) donor. Unless otherwise indicated, Cas9 ribonucleoproteins (RNPs) were assembled by complexing 100 pmol single guide RNA (sgRNA) with 2 μg recombinant SpCas9 per reaction. For electroporation, 244.4 fmol donor dsDNA was added to the pre-assembled RNP and applied to 5 × 10⁶ NK cells. The cytokine-stimulated NK cells were then electroporated using the Neon Transfection System (Thermo Fisher Scientific) with the indicated electroporation program settings. Following electroporation, NK cells were cultured in NK MACS (Miltenyi Biotec) supplemented with IL-2 (200 IU/mL, R&D Systems) and IL-15 (10 ng/mL; PeproTech), with or without 0.5 μM of dexamethasone (Dex; H0888, Sigma-Aldrich, St. Louis, MO, USA). The culture medium was partially refreshed on days 1, 2, and 4, and fully replaced (excluding Dex) on day 5.

For additional CAR KI at the NOXA (*PMAIP1*) locus, the donor dsDNA template was redesigned to include *PMAIP1* exon 2 region-specific homology arms flanking the CAR cassette. Cas9 RNP complexes were assembled using an NOXA-targeting sgRNA (spacer sequence: 5′-tcgagtgtgctactcaactc-3′). Genome editing at the NOXA cut site was evaluated via T7 endonuclease I (T7E1) assay and/or PCR-based genotyping using NOXA-specific primers. Targeted CAR integration was further confirmed via 5′ junction PCR followed by Sanger sequencing of the junction amplicon. All other experimental parameters, including donor DNA dose, electroporation program, and Dex exposure (0.5 μM), were identical to those used for TGFBR2 KI experiments. Primer sequences used for PCR amplification were as follows: T7E1 assay, forward 5′-ggctggtgacttatgctactc-3′ and reverse 5′-ccagcggtaatcttcggc-3′; 5′ junction PCR, forward 5′-ACA CTT GCC TCA TCC AGG-3′ and reverse 5′-GAG GGC CAT GGT GGC CTC GAG GAA TTC GAA CCC GGG CGA C-3′.

### Flow cytometry analysis

To assess TGFβRⅡ and CAR expressions in genetically engineered NK cells, cells were stained with eBioscience Fixable Viability Dye eFluor 780 (65-0865-18, Thermo Fisher Scientific) following the manufacturer's protocol to exclude dead cells. After two washes with PBS, cells were stained with APC-conjugated anti-human TGFβRⅡ antibody (Clone W17055E; 399706, BioLegend) and biotin-conjugated mesothelin antigen which binds the extracellular scFv domain of the mesothelin CAR, for 30 min at 4 °C. Following surface staining, cells were washed twice with PBS and incubated with phycoerythrin (PE)-conjugated streptavidin (554061, BD Biosciences, Franklin Lakes, NJ, USA) to detect mesothelin binding. After a final wash, samples were analyzed on a CytoFLEX flow cytometer (Beckman Coulter Life Sciences, Brea, CA, USA). Flow cytometry data were processed and analyzed using FlowJo software (BD Biosciences, San Jose, CA, USA).

### T7 endonuclease I (T7E1) assay

Genetically engineered NK cells were harvested, and genomic DNA was isolated using the EZ Genomic DNA Prep Kit (EP401-250N, Enzynomics, Daejeon, South Korea) according to the manufacturer's instructions. The target genomic region surrounding the CRISPR/Cas9 editing site was amplified by PCR, and the resulting amplicons were purified using the HiYield Plus™ Gel/PCR DNA Mini Kit (QDF300, Real Biotech Corporation, Taipei, Taiwan). For the T7E1 assay, purified PCR amplicons were denatured and re-annealed in NEBuffer 2 (NEB) using a thermocycler to allow the formation of mismatched heteroduplexes. The hybridized products were then digested with T7E1 (M0302L, NEB) at 37 °C for 30 min. Digested products were analyzed by electrophoresis on a 1% agarose gel, and band patterns were visualized to assess gene-editing efficiency. The primer sequences used in PCR amplification are as follows: T1/T2: Forward, 5′-TCACTCGCGCGCACG-3′ and Reverse, 5′- AGATAACCAACTTCTCAAACTTCCCTT-3′; T3/T4: Forward, 5′-GTCTGCTCCAGGTGATGTTTA-3′ and Reverse, 5′- GGGCCTGAGAATCTGCATTTA-3′; T5/T6: Forward, 5′- GCAGGGGATGACGAAC-3′ and Reverse, 5′-CTGCCCACTGTTAGCC-3′.

### Quantification of nuclear CAR KI donor DNA using Quantitative PCR (qPCR)

pNK cells were electroporated with CAR knock-in donor double-stranded DNA (dsDNA) containing the pSFFV promoter, poly(A) signal, and a glucocorticoid response element (GRE), and cultured with or without Dex. At 24 h post-electroporation, nuclei were isolated using a nuclear isolation kit (Nuclear Extraction Kit, ab113474, Abcam, Cambridge, UK), followed by genomic DNA extraction using EZ Genomic DNA Prep Kit (EP401-250N, Enzynomics). Nuclear donor DNA was quantified via qPCR using CAR-specific primers and normalized to GAPDH genomic DNA. The primer sequences used in PCR amplification are as follows: SS1-CAR: Forward, 5′- GAC GAC GCC ACC TAC TAC TG -3′ and Reverse, 5′- GCT AGC CTT GCA GCT GAT CT -3′; GAPDH genome region: Forward, 5′- GGT CTC CTC TGA CTT CAA CAG C -3′ and Reverse, 5′- AGA GTT GTC AGG GCC CTT TTT CT -3′.

### Western blot analysis

To assess pSMAD2/3 levels in NK cells, TGFβ1 (50 ng/mL; 781804, Biolegend) was treated to the NK cells and incubated for 3 h. Following stimulation, NK cells were lysed using RIPA buffer supplemented with cOmplete™ Protease Inhibitor Cocktail (11697498001, Sigma-Aldrich). Protein lysates were resolved by sodium dodecyl sulfate-polyacrylamide gel electrophoresis and transferred onto polyvinylidene fluoride membranes. Membranes were probed with the following primary antibodies: anti-pSMAD2/3 antibody (AP1343, ABclonal, Woburn, MA, USA), anti-SMAD2/3 antibody (8685, Cell Signaling Technology, Danvers, MA, USA), or anti-β-actin-horse radish peroxidase (HRP) (sc-47778 HRP, Santacruz, Dallas, TX, USA). After washing, membranes were incubated with HRP-conjugated Goat anti-Rabbit IgG (H+L) (31460, Thermo Fisher Scientific). Protein bands were visualized using the iDoc system (Bio-Rad, Hercules, CA, USA).

### Luminescence-based cancer-killing assay

To assess NK-mediated cytotoxicity, NanoLuc luciferase-expressing AsPC-1 cells (4×10^4^ cells/well) were seeded and cocultured with engineered NK cells at an E:T ratio of 1:1 for 24 h. After incubation, the co-cultures were washed with PBS to remove non-adherent cells and lysed using RIPA buffer (89901, Thermo Fisher Scientific). The resulting lysates were mixed with Nano-Glo Luciferase substrate (N1120; Promega, Madison, WI, USA), and the luminescence intensity was measured using a GloMax Microplate Reader (Promega). This assay enables quantification of viable target cancer cells based on retained luciferase activity, where decreased luminescence reflects increased NK cell-mediated cytotoxicity. To model TGFβ-rich immunosuppressive conditions, recombinant human TGFβ1 (781804, Biolegend) was added at the start of the NK-tumor co-culture and maintained throughout the assay. NK effector cells were co-cultured with target cells at the indicated E:T ratios for 24 h in the continuous presence of human TGFβ1 (20 ng/mL). Control conditions were performed in parallel without TGFβ1.

### CAR-normalized killing and post-KI Dex exposure

For CAR-normalized killing, CAR expression (%) was measured using flow cytometry immediately before co-culture, and the total effector NK input was adjusted so that the absolute number of CAR⁺ NK cells (CAR⁺ effector equivalents) was matched across one-step KI and one-step KI+Dex. For post-KI Dex exposure, KI NK cells were generated without Dex during manufacturing, then Dex (0.5 μM) was added after completion of KI during post-manufacturing culture for 24 h prior to the cytotoxicity assay. Cytotoxicity was assessed against AsPC-1 targets at E:T = 1:1 for 24 h as described above.

### CD107a degranulation assay

To evaluate NK cell degranulation activity, AsPC-1 cells (4×10^4^ cells/well) were co-cultured with NK cells at a 1:1 E:T ratio for 4 h. During the co-culture period, APC-conjugated anti-human CD107a (Clone H4A3; 560664, BD Biosciences) and Protein Transport Inhibitor (BD GolgiStop; 554724, BD Biosciences) were added according to the manufacturer's instructions. After incubation, cells were harvested and stained with FITC-conjugated anti-human CD56 (NCAM) antibody (Clone MEM-188; 304604, BioLegend) and PE/Cyanine7-conjugated anti-human CD69 antibody (Clone FN50; 310912, BioLegend) to assess NK cell activation status. Stained cells were analyzed by flow cytometry to quantify degranulation (CD107a expression) and activation (CD69 expression) within the CD56⁺ NK cell population.

### 5' junction PCR

To confirm site-specific CAR KI, CAR-engineered NK cells were harvested, and genomic DNA was extracted using the EZ™ Genomic DNA Prep Kit (Enzynomics) according to the manufacturer's instructions. PCR was performed using a 5' forward primer specific to the genomic region upstream of the targeted integration site and a 3' reverse primer specific to the inserted CAR transgene. The resulting PCR amplicons were analyzed by agarose gel electrophoresis and visualized using the iDoc Imaging System. The primer sequences used in 5' junction PCR are as follows: CAR: Forward, 5'-AAAAAACTGCAGCATTTCC-3' and Reverse, 5'-GCTTATCGTCGTCATCCTTG-3'; Actin: Forward, 5'-CACCATTGGCAATGAGCGGTTC-3' and Reverse, 5'-AGGTCTTTGCGGATGTCCACGT-3'.

### AAV production and titration

To produce AAV6 vectors carrying donor DNA for CAR KI, HEK293T cells were transfected with the pAAV6 plasmid (#6651 and #6665; Takara, Shiga, Japan) containing the donor DNA construct. Following transfection, the HEK293T culture supernatant was collected daily, and cells were harvested on day seven post-transfection. The collected supernatant was centrifuged at 1,500 rpm for 4 min to remove residual cells and debris. The clarified supernatant was supplemented with 0.1% (v/v) poloxamer 188 solution (P5556; Sigma-Aldrich) and concentrated using Pierce™ Protein Concentrators PES, 100K MWCO (Thermo Fisher Scientific). Simultaneously, harvested HEK293T cells were resuspended in PBS and subjected to three freeze-thaw cycles using liquid nitrogen. The lysed cell suspension was then centrifuged at 4,000 rpm for 5 min at 4 °C, and the resulting supernatant was collected. Both the concentrated supernatant and cell lysates were loaded onto an iodixanol gradient consisting of 15%, 25%, 40%, and 60% layers and prepared using OptiPrep™ 60% Iodixanol solution (Serumwerk, Bernburg, Germany) and 1 M NaCl/PBS-MK buffer (prepared with 5.84 g NaCl, 26.3 mg MgCl_2_, and 14.91 mg KCl in 100 mL PBS). Ultracentrifugation was performed at 200,000 x g for 2 h at 10 °C. The AAV-containing fraction was collected, and the buffer was exchanged to a formulation buffer containing 20 mM Tris-HCl (pH 7.4), 200 mM NaCl, 1 mM MgCl_2_, and 0.02% P188. For AAV titration, qPCR was performed with SYBR Green Master Mix (RT500M, Enzynomics) with the following primers: forward, 5'-GGAAACCCCTAGTGATGGAGTT-3'; reverse, 5'-CGGCCTCAGTGAGCGA-3'.

### Organoid preparation and modification

Patient-derived pancreatic cancer organoids were transduced with a lentiviral vector containing GFP and luciferase genes (pDRM210-LPG, Addgene #174722) to enable real-time tracking of viability. The transduced organoids were expanded in Cultrex® Reduced Growth Factor Basement Membrane Extract (BME; R&D Systems). For downstream assays, organoids were seeded at a volume of 20 μL per well in low-attachment Cellstar® 48-well plates (Greiner Bio-one, Monroe, NC, USA) and cultured under standard organoid growth conditions.

### Organoid and NK cell co-culture for cytotoxicity assessment

For cytotoxicity assays, patient-derived pancreatic cancer organoids stably expressing GFP and luciferase were seeded to Corning® 96-well Spheroid microplate (4520, Corning, NY, USA) without BME and added with NK cells at an E:T ratio of 2:1. After 24 h, apoptosis was assessed by adding CellEvent ™ Caspase-3/7 Red Detection Reagent (C10430; Thermo Fisher), and fluorescence images were obtained using a Nikon fluorescence microscope.

For quantification of organoid viability, D-luciferin (HY-12591B, MedChemExpress, Monmouth Junction, NJ, USA) was applied to each well, and luminescence was quantified using a Victor X3 plate reader (Revvity, Waltham, MA, USA). Relative cytotoxicity was calculated based on the reduction in luminescence compared to organoid-only control wells.

For flow cytometry-based analysis, organoid-NK cell co-cultures were dissociated using TrypLE™ Express Enzyme (12604013; Gibco). Cells were stained with Fixable Viability Dye eFlour™ 506 (65-0866-14; Thermo Fisher Scientific) to identify dead cells, followed by surface staining with anti-human EpCAM-APC (Clone 9C4; 324207, BioLegend) and anti-human CD56-PE (Clone HCD56; 318306, BioLegend). Dead organoid cells were defined as eFluor 506^+^ cells within the EpCAM^+^ population. Flow cytometry data were analyzed using FlowJo software (BD Bioscience).

### Library preparation for whole-exome sequencing (WES)

For library preparation of genetically engineered NK cell whole-exome sequencing, each sequencing sample is prepared according to the Illumina TruSeq DNA sample preparation guide to obtain a final library with an average insert size of 300-400 bp. Approximately 1 μg of genomic DNA is fragmented by Covaris systems to generate dsDNA fragments with 3' or 5' overhangs. The dsDNA fragments with 3' or 5' overhangs are converted into blunt ends using an end repair mix. The 3' to 5' exonuclease removes the 3' overhangs, and polymerases fill in the 5' overhangs. After end repair, fragments of the desired size were selected by adjusting the ratio of purification beads. To prevent fragments from ligating each other during the adapter ligation reaction, an adenine nucleotide is added to the 3' ends of the blunted fragments. This A-tailing step allowed ligation to adapters carrying a complementary 3′ thymidine overhang. Finally, multiple indexing adapters are ligated to the ends of the DNA fragments to prepare them for hybridization to the flow cell.

### On- and off-target profiling for CAR knock-in

WES was used to profile on-target integration and potential off-target insertions in WT and CAR-NK cells. Paired-end reads were aligned to the GRCh38 human reference genome using the iSAAC aligner (version iSAAC-04.18.11.09) 27. During alignment, low-quality bases at the 3′ end and adapter sequences were trimmed, and the resulting alignments were output as sorted, duplicate-marked BAM files. Additionally, reads with more than 20% of bases having a Phred quality score < 30 were removed to ensure high-confidence variant detection. To evaluate genome editing outcomes, candidate off-target sites were identified using Cas-OFFinder with up to three mismatches permitted relative to the sgRNA target sequence 28. Reads at the on-target locus and predicted off-target regions were visually inspected using Integrative Genomics Viewer (IGV; version 2.29.2) 29. Chimeric reads spanning the junction between genomic DNA and the CAR construct were considered evidence of targeted insertion.

### RNA extraction, library preparation, and sequencing

Total RNA was isolated from human pNK cells using RiboEx™ Total RNA isolation solution (GeneAll, South Korea) in accordance with the manufacturer's protocols. Libraries for 150bp paired-end sequencing were constructed using the TruSeq Stranded mRNA Sample Prep Kit (Illumina, CA, USA). Specifically, mRNA molecules were purified and fragmented from 0.1-1 μg of total RNA using oligo(dT) magnetic beads. The fragmented mRNA was reverse-transcribed into first-strand cDNA using random hexamer primers, followed by second-strand synthesis to generate double-stranded cDNA. After sequential end repair, A-tailing, and adapter ligation processes, the cDNA libraries were amplified by PCR. The quality of constructed cDNA libraries was assessed using the Agilent 2100 BioAnalyzer (Agilent Technologies, CA, USA). Quantification was performed using the KAPA library quantification kit (Kapa Biosystems, MA, USA) according to the manufacturer's library quantification protocol. After denaturation and cluster generation, paired-end sequencing (2 × 150 bp) was carried out on the Illumina NovaSeq X Plus platform (Illumina).

### RNA-seq data processing

To enhance read quality, raw FASTQ data were cleaned using Trimmomatic (version 0.39) 30. Trimmed data were aligned to the GRCh38 reference genome using STAR aligner (version 2.7.11b), and gene and transcript quantification was performed with RSEM (version 1.3.3) 31, 32. To correct for batch effects identified in the dataset, expected counts from RSEM were adjusted using the negative binomial regression model implemented in ComBat-Seq (sva package, version 3.52.0) 33. Adjusted counts were further processed to remove genes with consistently low expression across samples. Normalization factors were computed using the trimmed mean of M-values (TMM) method implemented in edgeR (version 4.2.2) and adjusted counts were normalized accordingly 34. Subsequently, normalized counts were transformed into log2 counts per million (log2CPM) using the voom function in limma 35 (version 3.60.6). Differential expression analysis between samples was performed using linear model fitting provided by limma voom. Genes with absolute log2 fold change ≥ 0.5 and adjusted *p*-values < 0.05 were defined as differentially expressed genes (DEGs). In addition, Gene Set Enrichment Analysis (version 4.1.0) was performed, setting cytotoxicity as a continuous phenotype variable 36. Gene sets with an adjusted *p*-value < 0.05 were considered statistically significant.

### Library preparation and sequencing for ATAC-seq

Library construction was performed using ATAC-Seq Library Prep Kit for Illumina (Active Motif, USA) according to the manufacturer's instructions. Briefly, a total of 100,000 fresh cells were washed twice with 100 μl of cold PBS, resuspended in 100 μl of lysis buffer, and nuclei were prepared on ice for 10 min. Immediately after cell lysis, the nuclear suspension was centrifugated at 500×g at 4 ℃ for 10 min and the supernatant was removed. The nuclei were then incubated with 50 μl of Tagmentation Master Mix at 37℃ for 30 min in a thermomixer set at 800 rpm. DNA was purified using DNA purification column in kit. PCR was performed to amplify the library for 10 cycles using the following PCR conditions : 72 ℃ for 5 min; 98 ℃ for 30 s; and thermocycling at 98 ℃ for 10 sec, 63 ℃ for 30 s and 72 ℃ for 1 min. The library was purified using SPRI bead (Beckman Coulter) to remove contaminating primer dimers. Library quantity was verified using TapeStation HS D5000 Screen Tape (Agilent Technologies). Last all libraries were sequenced on the NovaSeq 6000 with 100 bp paired-end reads (Illumina).

### ATAC-seq data processing

To remove adapters, low-quality sequences, poly-G sequences, and excessively short reads, raw sequencing reads underwent quality filtering and adapter trimming using both Trimmomatic (version 0.39) and fastp 30, 37 (version 0.12.1). Filtered reads were aligned to the GRCh38 reference genome using Burrows-Wheeler Aligner (BWA) mem (version 0.7.19 (r1273)), followed by the removal of mitochondrial DNA (mtDNA), PCR duplicates, and non-uniquely mapped reads using samtools 38, 39 (version 1.21). To precisely identify accessible chromatin regions, Tn5 insertion sites were adjusted using deepTools 40 (version 3.5.6). Peaks representing open chromatin regions were called with MACS2 (version 2.2.9.1), and regions overlapping with the ENCODE blacklist were excluded 41, 42. For downstream analysis, fragments corresponding to nucleosome-free regions were selected using bamCoverage (version 3.5.6) with the parameters --minFragmentLength 0 and --maxFragmentLength 150 40. To compare chromatin accessibility around transcriptional start sites (TSSs) of genes, signal intensities from bigWig files were summarized using deepTools computeMatrix (version 3.5.6). The resulting matrices were used to visualize accessibility at TSSs across the genome, as well as at TSSs of TGF-β signaling pathway genes (KEGG PATHWAY ID: hsa04350), DEGs and non-DEGs identified from the comparison between WT and WT-Dex, using deepTools plotHeatmap and plotProfiles. Differential chromatin accessibility analysis was performed using DESeq2 (version 1.44.0), and peaks with an absolute log2 fold change ≥ 0.5 and adjusted *p*-value < 0.05 were defined as statistically significant 43. Differentially accessible regions were manually inspected through the UCSC Genome Browser, and corresponding motif-associated gene annotations were performed using ChIPseeker 44, 45 (version 1.40.0).

### qRT-PCR validation of candidate genes

Total RNA was isolated from IL-12, IL-15, and IL-18 stimulated WT and WT+Dex NK cells at day 7 post Dex exposure using TRIzol™ Reagent (Thermo fisher scientific) according to the manufacturer's instructions. cDNA was synthesized from 1 μg RNA using TOPscript™ RT DryMIX (dT18) (Enzynomics). Quantitative PCR was performed on a QuantStudio 3 system (Applied Biosystems) using TOPreal™ SYBR Green qPCR PreMIX (Enzynomics) with gene-specific primers for IGF1, GLUL, and PIK3IP1. Relative expression levels were calculated using the ΔΔCt method, normalized to GAPDH, and expressed as fold change relative to WT controls. The primer sequences used in PCR amplification are as follows: GAPDH mRNA: Forward, 5'-CAG CCT CAA GAT CAT CAG CA-3' and Reverse, 5'-TGT GGT CAT GAG TCC TTC CA-3'; IGF1: Forward, 5'-AGA GCC TGC GCA ATG GAA TA-3' and Reverse, 5'-GAG ATG CGA GGA GGA CAT G -3'; GLUL: Forward, 5'-GGA GAA GAG CGG AGC GTG-3' and Reverse, 5'-ATG GTG GAA GGT GTT CTG GT-3'; PIK3IP1: Forward, 5'-CCA GTG ATT GGG ATC AGC CA-3' and Reverse, 5'-AGC TCC GAT GGC AAT GAT GA-3'.

### Mouse xenograft studies

To establish a subcutaneous pancreatic cancer xenograft model, MSLN-overexpressing AsPC-1 cells were suspended in PBS, then mixed with Matrigel (Corning) at a 1:1 ratio. A total of 3 × 10⁶ cells in 100 μL were injected subcutaneously into the right flank of 6-week-old male NOD-Prkdc^em1Baek^Il2rg^em1Baek^ (NSG) mice (JA BIO, Suwon, South Korea). When tumors became established (day 7 post-inoculation), mice were randomized into four groups: cancer only, mock, one-step KI, and one-step KI+Dex. Mock NK cells were generated by electroporation without sgRNA or donor DNA. One-step KI NK cells were generated by electroporation-mediated TGFBR2 KO and CAR KI without Dex, whereas TGFBR2-KO CAR KI plus Dex NK cells were generated using the same manufacturing workflow with the addition of Dex (0.5 μM) during the manufacturing phase, as described above. NK cells were administered intravenously via tail vein injection on days 11, 13, and 15 at a dose of 1 × 10⁷ cells per mouse in 100 μL PBS supplemented with 10,000 IU IL-2 per mouse. Tumor size was measured three times a week using a caliper, and tumor volume was calculated using the formula (length × width × height). Body weight was monitored regularly throughout the study. At the experimental endpoint (day 31 post-tumor inoculation), tumors were excised, photographed, and weighed. For histological analysis, excised tumors were fixed in 4% paraformaldehyde in PBS for 24 h, embedded in paraffin, sectioned at 4 μm, and stained with hematoxylin and eosin (H&E) following standard procedures.

### Seahorse metabolic flux analysis

Mitochondrial respiration and glycolytic activity of NK cells were quantified via extracellular flux analysis using a Seahorse XFe analyzer (Agilent Seahorse XFe96, Agilent Technologies). pNK cells were prepared in four conditions: WT, WT+Dex, KI, and KI+Dex, generated as described above. For Dex-treated groups, 0.5 μM of Dex was included during the manufacturing period. On the day of assay, Seahorse cell culture microplates were coated with poly-D-lysine, according to the manufacturer's instructions to facilitate NK-cell adherence. NK cells were washed and resuspended in Seahorse XF assay medium (Agilent Technologies) supplemented with 10 mM glucose, 1 mM pyruvate, and 2 mM glutamine (pH 7.4), and plated at 4 × 10^5^ cells/well in a final volume of 180 μL. Plates were incubated for 60 min at 37 °C in a non-CO₂ incubator prior to measurement. For mitochondrial respiration and glycolytic activity, oxygen consumption rate (OCR) and extracellular acidification rate (ECAR) were measured using the Seahorse XF Cell Mito Stress Test. Basal OCR was recorded followed by sequential injections of oligomycin (1.5 μM), FCCP (1.0 μM), and rotenone/antimycin A (0.5 μM each) to determine ATP-linked respiration, maximal respiration, and non-mitochondrial respiration. OCR and ECAR values were calculated using Seahorse Wave software (Agilent).

### Droplet digital PCR (ddPCR) for CAR copy number in CAR-NK^KO/KI^ genomic DNA

Genomic DNA (gDNA) from CAR-NK^KO/KI^ cells was subjected to droplet digital PCR (ddPCR) using a QX200 Droplet Digital PCR system (Bio-Rad) to quantify the absolute abundance of the CAR sequence (SS1) relative to a genomic reference (TGFBR2 exon 4, sgRNA T6 target flanking region). ddPCR reactions (total volume 20 μL) were prepared using ddPCR Supermix for Probes (no dUTP) (Bio-Rad) at 1×, primer/probe mix at 250 nM, and 30 ng gDNA input per reaction. Duplex detection was performed using a FAM-labeled SS1 (CAR) probe and a HEX-labeled TGFBR2 probe. Droplets were generated using Bio-Rad droplet generation oil for probes and subsequently amplified on a C1000 Touch Thermal Cycler (deep-well) (Bio-Rad) with the following cycling conditions: 95 °C for 10 min; 40 cycles of 94 °C for 30 s and 60 °C for 1 min (ramp approximately 2 °C/sec); 98 °C for 10 min; and 4 °C for 30 min/hold. After PCR, droplets were read on a QX200 droplet reader (Bio-Rad), and data were analyzed using QuantaSoft software using Poisson-based absolute quantification. The primer and probe sequences used in PCR amplification are as follows: ddPCR-SS1-CAR: Forward, 5′-CAC CAT GAA CTG GGT GAA G-3′, Reverse, 5′-GCC TCT GAA CTT CTG ATT GTA G-3′, and Probe, 5′-6-FAM-ATC CAC TCC AGG CTC TTG CC-Black Hole Quencher 1-3′; ddPCR-TGFBR2: Forward, 5′-CGT GTG CCA ACA ACA TCA AC-3′, Reverse, 5′- TGC TTC AGC TTG GCC TTA TAG-3′, and Probe, 5′-HEX-CCA TTG AGC TGG ACA CCC TGG T-Black Hole Quencher 1-3′.

### Statistical analysis

All statistical analyses were performed using GraphPad Prism 10. Unless otherwise stated, biological replicates represent one representative NK donors. Data are presented as mean ± SD, as indicated in the corresponding figure legends. For statistical analysis between two groups, we used Student's T-test. For comparisons involving three or more groups, we used one-way ANOVA followed by Tukey's post-hoc multiple-comparisons test, unless a single control comparison was specified, in which case Dunnett's post-hoc test was used. For *in vivo* tumor growth curves, we used two-way ANOVA followed by Tukey's post-hoc multiple-comparisons. Statistical significance was set at a *p*-value < 0.05. For RNA-seq and ATAC-seq, differential gene expression or differential chromatin accessibility was assessed using linear model fit provided by limma voom or DESeq2.

## Results

### sgRNA selection for *TGFBR2* knock-out (KO)

**Figure 1:**
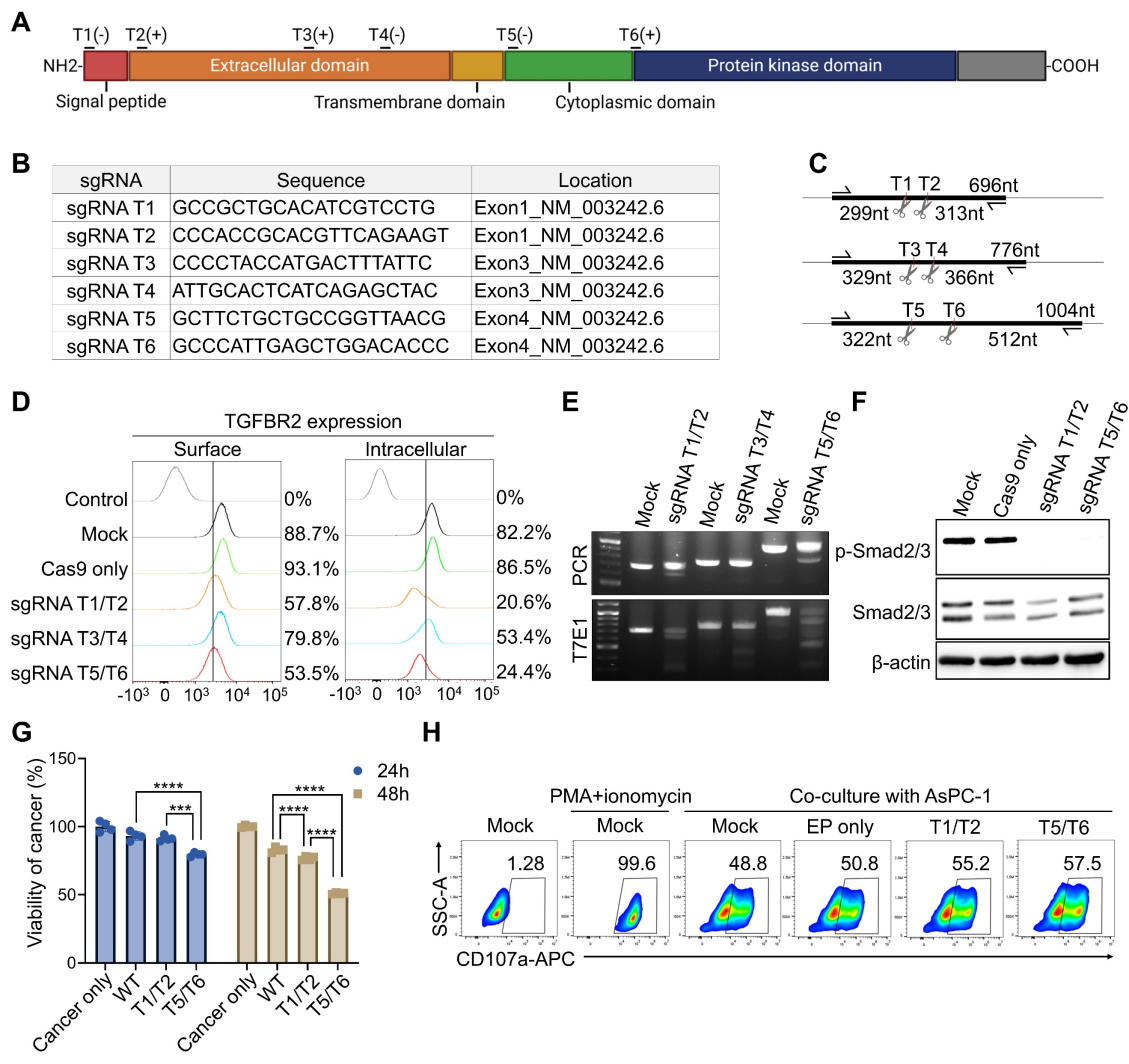
** single guide RNA (sgRNA) selection for *TGFBR2* knock-out (KO). (A)** Schematic representation showing the target locations of candidate sgRNAs within the *TGFBR2* gene. **(B)** Sequences and genomic positions of each sgRNA candidate. **(C)** Predicted DNA fragment sizes based on PCR and T7E1 assays following KO with each sgRNA pair. **(D)** Flow cytometric analysis of TGFβRII expression on the surface and intracellular compartments of primary NK (pNK) cells following electroporation with indicated sgRNAs. For sgRNA screening, each sgRNA pair (T1/T2, T3/T4, and T5/T6) was delivered simultaneously as a dual-guide Cas9 ribonucleoproteins (RNP) combination. The control histogram represents isotype-matched control staining. Percentages indicate the proportion of TGFβRII-positive cells relative to the isotype control. Summary data of surface TGFβRII expression percentages and MFI values are provided in Supplementary Table 1. **(E)** T7E1 assay results demonstrating editing efficiency of each sgRNA pair. **(F)** Phospho-Smad2/3 levels detected in KO pNKs following TGFβ1 treatment. **(G)** Viability of AsPC-1 cancer cells co-cultured with KO pNK cells following TGFβ1 treatment. WT refers to Cas9-only-electroporated NK cells. **(H)** CD107a expression on KO pNK assessed after co-culture with AsPC-1 cells.* p*-values were calculated using one-way ANOVA with Tukey's multiple-comparisons test. Data are shown as mean ± SD. Representative data from one NK donor is shown; The experiment was independently repeated using NK cells from additional donors with comparable results. Summary data are provided in Supplementary Table 1. *** *p* < 0.001 and **** *p* < 0.0001.

To identify the optimal sgRNA sequence for CRISPR-Cas9-mediated KO of *TGFBR2* gene in pNK cells, and to ensure compatibility with subsequent HDR-based KI strategies, we designed six different sgRNA pairs targeting various regions of the gene (Figure 1A). The sgRNA pairs were grouped based on their target exons: T1 and T2 target exon 1, T3 and T4 target exon 3, and T5 and T6 target exon 4 (Figure 1B). Exon 4 was selected as a candidate locus because it was intended as the CAR KI site in downstream experiments. Predicted DNA fragment sizes following PCR and T7E1 assays post-KO have been illustrated (Figure 1C). To assess KO efficiency in cytokine-activated pNK cells, each sgRNA pair was delivered simultaneously as a dual-guide Cas9 RNP combination via electroporation, enabling generation of larger deletions between the two cut sites. TGFβRⅡ expression was subsequently analyzed on both the cell surface and in intracellular compartments. All three pairs induced *TGFBR2* KO; however, the T1/T2 and T5/T6 pairs resulted in a more pronounced reduction in TGFBR2 expression compared with the T3/T4 pair (Figure 1D). Consistently, the T7E1 assay confirmed that higher indel frequencies with the T1/T2 and T5/T6 pairs (Figure 1E). Functionally, following *TGFBR2* KO using either the T1/T2 or T5/T6 pairs, phosphorylated SMAD2/3 (pSMAD2/3) was no longer detectable in pNK cells treated with TGFβ1, confirming successful pathway disruption (Figure 1F). To evaluate the impact on NK cell cytotoxicity, *TGFBR2-*KO pNK cells were co-cultured with the PDAC cell line AsPC-1, and cancer cell killing activity was assessed. Notably, *TGFBR2 KO* resulted in a significant enhancement of NK cell-mediated cytotoxicity, with the T5/T6 pair showing the most substantial effect (Figure 1G). This finding correlated with increased expression of the degranulation marker CD107a in pNK cells following co-culture (Figure 1H). Based on combined molecular, signaling, and functional readouts, the T5/T6 sgRNA pair was selected as the lead configuration for subsequent optimization toward the KI workflow. Hence, the T5/T6 sgRNA pair most efficiently disrupted TGFβRⅡ*,* and *TGFBR2-*KO significantly enhanced the cancer cell killing activity of pNK cells.

### Optimization of Cas9-RNP conditions for electroporation-mediated KI

**Figure 2:**
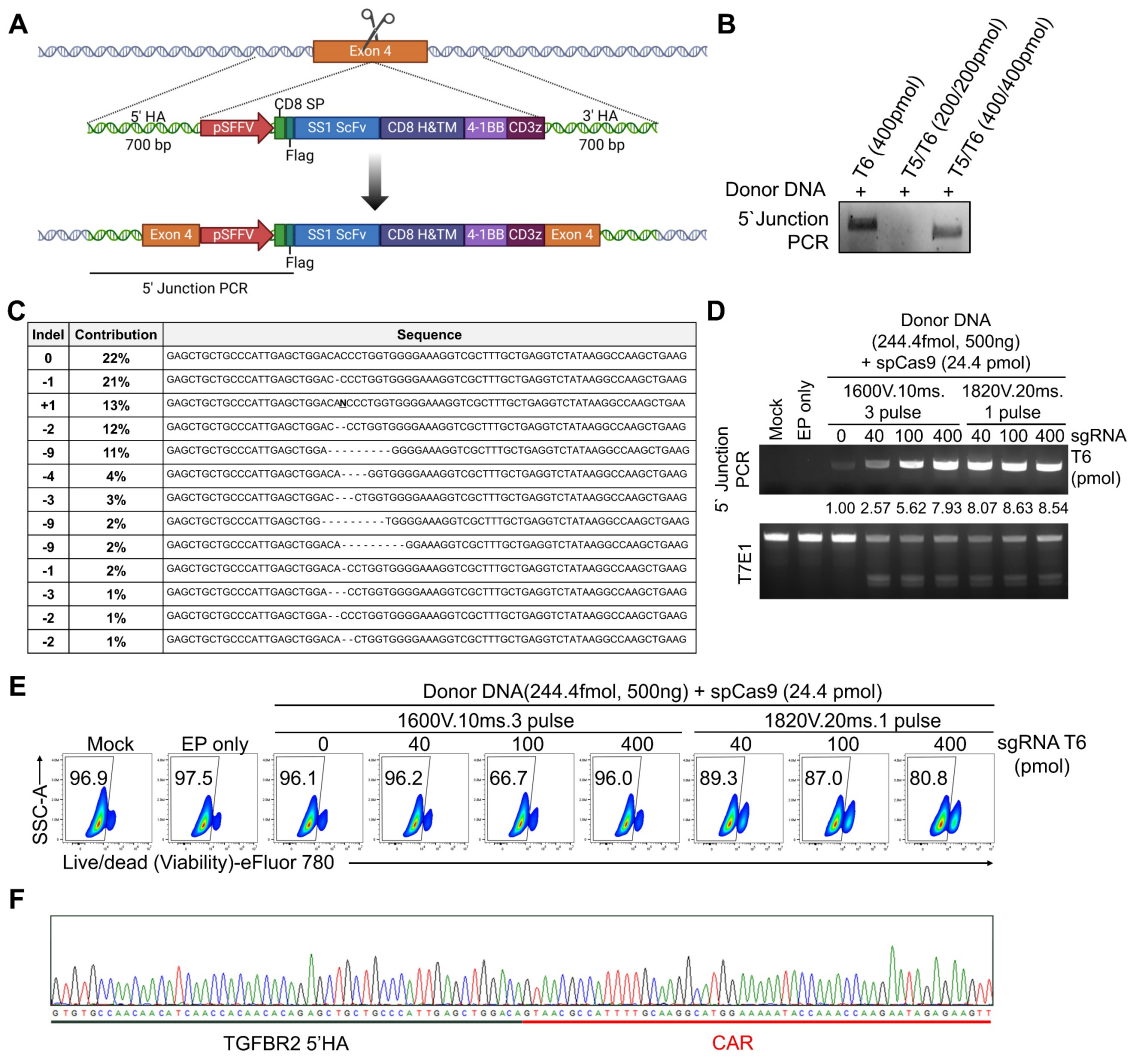
** Optimization for electroporation-mediated KI. (A)** Schematic diagram of the chimeric antigen receptor (CAR) KI strategy at the *TGFBR2* locus using homology-directed repair (HDR). **(B)** 5' Junction PCR analysis of pNK cells following electroporation with donor DNA, spCas9 and either T5/T6 sgRNA pair or T6 alone. **(C)** Representative indel sequences observed after *TGFBR2* KO.** (D)** 5' Junction PCR and T7E1 assay results following electroporation with donor DNA, spCas9 and sgRNA T6. **(E)** Viability of pNK cells following electroporation, as assessed by flow cytometry. **(F)** Sanger sequencing results confirming precise *TGFBR2* KO and CAR KI at the target locus.

To generate *TGFBR2*-KO CAR-NK (CAR-NK^KO/KI^) cells, we designed a donor dsDNA construct containing a CAR-expressing cassette flanked by 5' and 3' homology arms matching the *TGFBR2* gene. This donor was intended to be inserted into the *TGFBR2* locus via HDR following CRISPR/Cas9-induced double-strand break (DSB), enabling simultaneous disruption of TGFβRⅡ and expression of the CAR construct (Figure 2A). Having identified T5/T6 as the most efficient sgRNA pair for TGFBR2 KO, we next optimized the conditions for HDR-mediated CAR KI at TGFBR2 exon 4. We directly compared dual-guide (T5/T6) versus single-guide (T6 only) delivery in the KI setting under otherwise identical electroporation and donor DNA conditions. PCR analysis of the 5' junction between *TGFBR2* exon 4 and the CAR cassette revealed that sgRNA T6 alone mediated the most efficient KI (Figure 2B). Sanger sequencing confirmed the presence of indel mutations at exon 4 induced by sgRNA (Figure 2C). Hence, we used sgRNA T6 for subsequent KI experiments and optimized electroporation conditions and sgRNA concentrations. Two electroporation protocols were tested: 1600 V, 10 ms, 3 pulses versus 1820 V, 20 ms, 1 pulse. The latter protocol showed higher KI efficiency (Figure 2D). However, cell viability dropped below 90% with 1820 V, 20 ms, and 1 pulse, and further decreased as the sgRNA concentration increased (Figure 2E). Integration of the donor DNA into *TGFBR2* exon 4 was confirmed by sequencing the genomic DNA of KI NK cells, where the CAR cassette sequence was identified at the target locus (Figure 2F and Figure S1). Additionally, genome-wide assessment using whole-exome sequencing revealed no chimeric reads containing the CAR sequence across seven predicted off-target sites, supporting the targeting specificity of the designed sgRNA (Figure S1B-C). Therefore, for the final one-step KO/KI manufacturing workflow, we selected T6 as a single guide for *TGFBR2* targeting and used this configuration in all subsequent experiments. In summary, we successfully established optimal electroporation parameters and sgRNA conditions for generating *TGFBR2-*disrupted CAR-NK cells through a one-step process, termed “one-step CAR-NK^KO/KI^.”

### Optimization of donor dsDNA constructs to enhance KI efficacy

**Figure 3:**
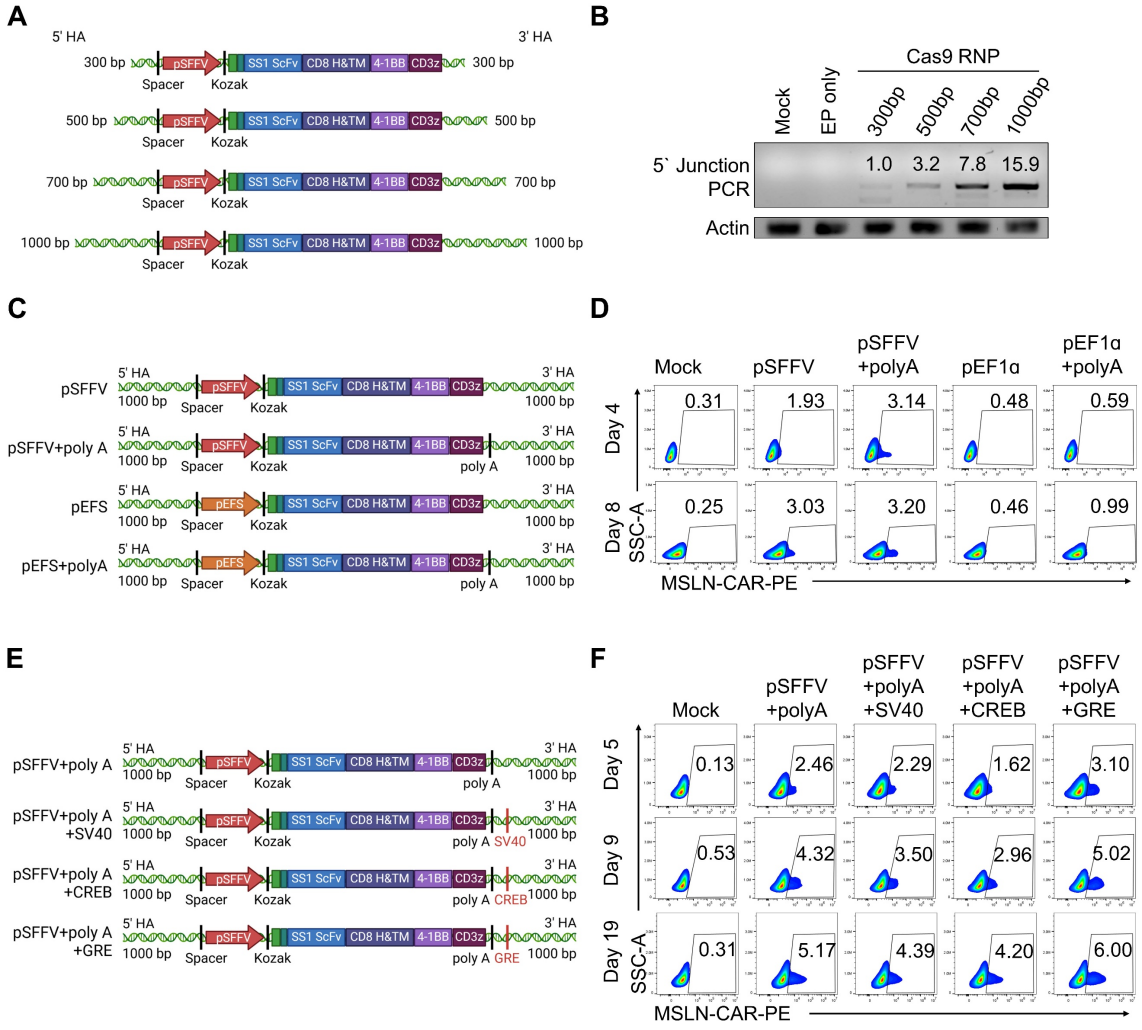
** Optimization of donor DNA for electroporation-mediated KI. (A)** Schematic diagram of the CAR KI strategy using donor DNAs with various homology arm (HA) lengths. **(B)** 5' Junction PCR analysis of CAR KI efficiency using donor DNAs with HA lengths. **(C)** Schematic representation of CAR expression constructs incorporating different promoters and polyadenylation [poly(A)] signals. **(D)** CAR expression levels assessed using flow cytometry following KI with each CAR construct. CAR expression was detected via staining with biotin-conjugated mesothelin antigen followed by PE-conjugated streptavidin, which binds the extracellular scFv domain of the mesothelin CAR.** (E)** Schematic diagram of CAR donor constructs containing various DNA nuclear targeting sequences (e.g., SV40, CREB, GRE). **(F)** CAR expression levels following KI using donor DNAs incorporating different nuclear targeting sequences.

To further improve HDR-mediated KI efficiency, we redesigned the donor dsDNA construct. Initially, we investigated the effect of homology arm (HA) length by varying both the 5' and 3' HAs from 300 to 1000 bp (Figure 3A). We observed a positive correlation between HA length and KI efficiency, with the 1000 bp arms yielding the highest efficiency (Figure 3B). Hence, 1000 bp HAs were adopted for all subsequent experiments. To enhance CAR gene expression post-integration, we replaced the upstream EF1α short (pEFS) promoter with the spleen focus-forming virus promoter (pSFFV) and a polyadenylation (poly-A) tail downstream of the CAR coding region (Figure 3C). Flow cytometry analysis of CAR expression following KI of four different donor constructs revealed that the highest expression levels with the SFFV + poly-A combination (Figure 3D).

Next, we optimized electroporation conditions for the redesigned donor construct. Specifically, we introduced a 500 V, 100 ms, 1 pulse step following the standard protocol of 1820 V, 20 ms, 1 pulse. This sequential electroporation protocol enhanced KI efficiency, as indicated by increased 5' junction PCR signal intensity. T7E1 analysis also showed improved *TGFBR2-*KO efficiency (Figure S2A). Consistently, CAR expression increased while TGFβRⅡ expression was significantly reduced under this optimized condition (Figure S2B). To further augment HDR-mediated KI efficiency, we hypothesized that enhancing cytoplasmic trafficking and nuclear accumulation of donor DNA could improve CAR integration. Accordingly, we appended transcription factor (TF)-binding DNA regulatory motifs-including the simian vacuolating virus 40 binding element (SV40), cyclic AMP response element-binding protein binding element (CREB), and glucocorticoid response element (GRE)— to the ends of the donor DNA 46-50 (Figure 3E). These elements were selected based on prior studies describing their ability to facilitate receptor- or TF-mediated nuclear trafficking of plasmid DNA, rather than functioning as classical nuclear localization signals. Among the tested motifs, the GRE significantly boosted CAR expression, consistent with enhanced nuclear accumulation of donor DNA via GR-mediated shuttling, consequently improved KI efficiency (Figure 3F). Importantly, KI efficiency varied depending on the target locus rather than being uniformly low across the genome. When targeting an alternative locus, NOXA, an apoptosis-related gene, using a similar non-viral KI strategy with a GRE-containing donor construct, we observed a higher average KI efficiency of approximately 7.6% in pNK cells (Figure S3). The NOXA locus was selected based on our previous report showing that NOXA knockout enhances NK cell-mediated cytotoxicity 51. These data indicate that KI efficiency in pNK cells is strongly influenced by genomic context. In conclusion, we established an optimized “one-step CAR-NK” for simultaneous *TGFBR2-*KO and CAR KI. This method integrated dsDNA containing 1000 bp homology arms, the SFFV promoter, a poly-A tail, and a GRE sequence, along with a dual-pulse electroporation protocol consisting of 1820 V for 20 ms (1 pulse) followed by 500 V for 100 ms (1 pulse). This approach significantly enhances both HDR-mediated knock-in and CAR expression in pNK cells.

### Dex treatment enhanced the CAR KI efficiency and NK cytotoxicity

**Figure 4:**
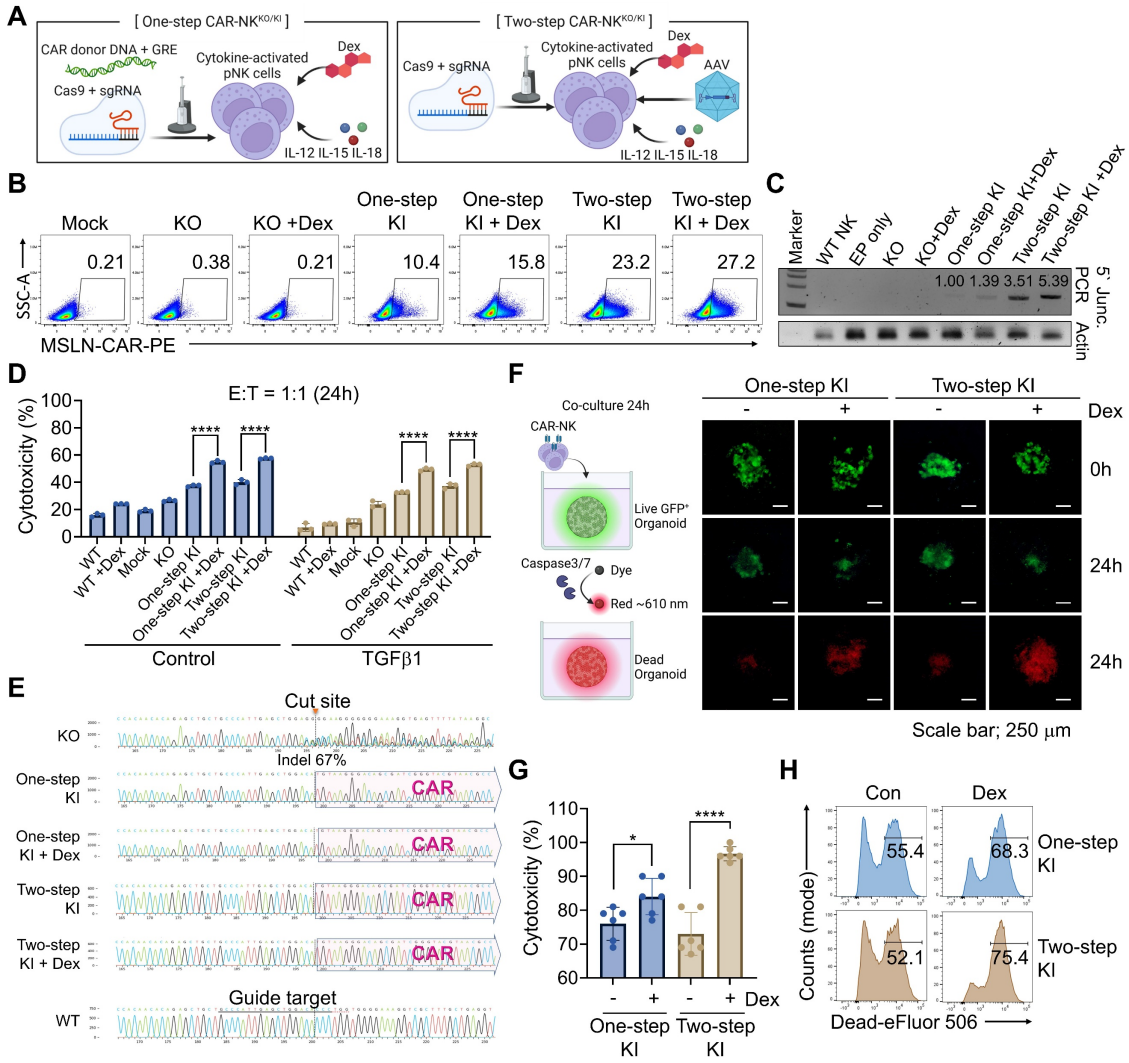
** Dexamethasone (Dex) enhances CAR KI efficiency and NK cell function**. **(A)** Schematic representation of the one-step and two-step CAR-NK cell manufacturing workflows. **(B)** CAR expression levels assessed by flow cytometry following KI using one-step or two-step procedures, with or without Dex treatment. **(C)** 5′ junction PCR analysis confirming KI efficiency across different manufacturing conditions. **(D)** Cytotoxicity of engineered NK cells evaluated after co-culture with AsPC-1 cells in the presence of TGFβ1. WT denotes cytokine-induced pNK cells without electroporation, and Mock denotes pNK cells subjected to Cas9-only electroporation without sgRNA or donor DNA. Data are presented as mean ± SD. Statistical significance was assessed using one-way ANOVA followed by Tukey's multiple-comparisons test; **** *p* < 0.0001. **(E)** Representative Sanger sequencing confirming site-specific CAR knock-in at the TGFBR2 locus. **(F)** Representative fluorescence images of Caspase 3/7 activation (red) in GFP-expressing patient-derived cancer organoids (green) co-cultured with CAR-NK cells. **(G)** Quantification of cancer organoids killing using a luminescence-based assay. **(H)** Flow cytometry analysis of organoid cell death using viability dye within the EpCAM^+^ population. WT, unedited pNK cells cultured without Dex; WT+Dex, unedited pNK cells cultured with 0.5 μM Dex; Mock, pNK cells subjected to electroporation with Cas9 but without sgRNA and donor DNA; KI ± Dex, TGFBR2-knockout CAR knock-in pNK cells generated with or without Dex during manufacturing.

We demonstrated that electroporation-mediated delivery of Cas9 RNPs and GRE-containing CAR donor DNA significantly enhanced KI efficiency (Figure 3). To further optimize this process, we compared an electroporation-based one-step CAR-NK^KO/KI^ manufacturing approach with a two-step approach involving AAV-mediated donor DNA delivery. Given that the GRE sequence, which responds to Dex, was included in the donor DNA to promote nuclear import, we investigated whether Dex treatment during CAR-NK^ KO/KI^ production could further enhance KI efficiency (Figure 4A). To directly assess whether Dex influences donor DNA nuclear accumulation, we isolated nuclear DNA 24 h after electroporation in the presence or absence of Dex, and quantified donor-specific sequences using qPCR. Dex treatment substantially increased the nuclear copy number of donor DNA across independent donors, whereas negligible signals were detected in negative control samples (Figure S4). These results indicate that Dex exposure enhances nuclear availability of the donor DNA, particularly in the context of the GRE-containing construct.

For CAR-NK^KO/KI^ cell generation, pNK cells were activated for 7 h in a cytokine cocktail containing IL-12, IL-15, and IL-18 52, 53. The activated NK cells were then electroporated with spCas9, sgRNA, and donor GRE-containing dsDNA, followed by incubation in NK MACS medium supplemented with IL-2 and IL-15, with or without 0.5 μM of Dex. In the two-step protocol, AAV-delivered donor DNA was added 30 min post-electroporation with Cas9 RNPs. Media was partially replaced on days 1, 2, and 4, and fully replaced (excluding Dex) on day 5 (Figure S5A). To evaluate KI efficiency, we assessed both CAR expression and genomic integration of donor DNA in both one-step and two-step CAR-NK cells. Remarkably, flow cytometry revealed that DEX treatment increased CAR expression in both approaches (Figure 4B). These findings were corroborated by 5' junction PCR, showing enhanced KI efficiency in DEX-treated groups (Figure 4C). These results indicate that Dex exposure enhances nuclear availability of the donor DNA, particularly in the context of the GRE-containing construct. To further distinguish whether the GRE/Dex-associated increase reflected enhanced transcription from pre-existing integrations or an increase in allele-level knock-in, we performed genomic copy-number analysis of the CAR cassette relative to the TGFBR2 locus. This analysis revealed an approximately 1.39-fold increase in CAR copy number in Dex-treated KO/KI samples compared with untreated controls (Figure S6), closely mirroring the magnitude of Dex-associated increases in CAR-positive frequencies observed via flow cytometry. These data indicate that Dex conditioning enhances CAR knock-in at the allele level, rather than solely augmenting transcription from existing integrations.

We assessed the cytotoxic function of CAR-NK^ KO/KI^ cells using MSLN-overexpressing AsPC-1 cells expressing nanoluciferase. Co-culture at a 1:1 ratio for 24 h in the presence of TGFβ1 revealed that *TGFBR2*-KO NK cells exhibited improved cytotoxicity compared to unstimulated controls, an effect further augmented by CAR expression (Figure 4D). Notably, Dex-treated CAR-NK^KO/KI^ cells displayed significantly enhanced cancer cell killing. To determine whether this enhancement was solely attributable to an increased frequency of CAR⁺ NK cells, we performed additional analyses in which cytotoxicity was normalized to the proportion of CAR⁺ effector cells. Dex-treated CAR-NK cells retained superior killing activity after CAR normalization. In addition, Dex administration after completion of the KI process further enhanced cytotoxicity, indicating that Dex augments NK cell function beyond its effect on CAR KI efficiency alone (Figure S7). Genomic analysis confirmed successful *TGFBR2*-KO (67% indel rate in the KO-only group) and CAR integration into the *TGFBR2* locus in both one- and two-step CAR-NK^KO/KI^ cells (Figure 4E and Figure S5B). Furthermore, cancer organoid assays showed increased caspase-3/7 activity and enhanced cytotoxicity in Dex-enhanced CAR-NK^KO/KI^ cells (Figure 4F and Figure S8, S9). These results were further validated using luminescence-based cytotoxicity assays (Figure 4G), and flow cytometry with live/dead staining, confirming superior killing efficacy (Figure 4H). To directly demonstrate functional inhibition of TGFβ signaling, we assessed canonical SMAD2/3 phosphorylation in pancreatic cancer organoid co-culture systems. As shown in Figure S10, TGFβ1-induced SMAD2/3 phosphorylation was markedly reduced in TGFBR2-KO CAR-NK cells compared with WT controls across independent organoid donors. These data confirm that TGFBR2 KO effectively suppresses downstream TGFβ signaling in a physiologically relevant organoid context. Together, these results demonstrate that the one-step KO/KI strategy, particularly when combined with Dex conditioning, enables efficient CAR integration, robust NK cell cytotoxicity, and functional resistance to TGFβ-mediated immunosuppression, supporting its therapeutic potential for solid tumor immunotherapy.

### Dex augmented NK cell cytotoxicity through metabolic reprogramming

**Figure 5:**
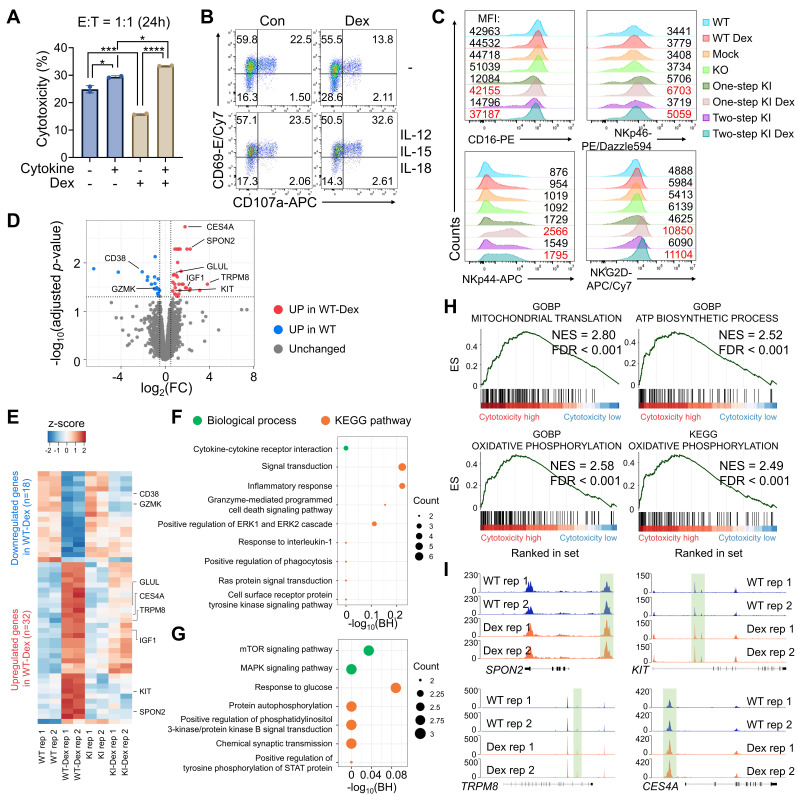
** Transcriptomic and chromatin accessibility profiling reveal Dex-mediated modulation of NK cell function. (A)** Cytotoxicity assay against AsPC-1 cells using unstimulated or IL-12/15/18-activated NK cells treated with or without Dex. **(B)** Expression levels of CD107a and CD69 on NK cells under unstimulated or cytokine-activated conditions following co-culture with AsPC-1 cancer cells, with or without Dex. **(C)** Surface expression of activating NK cell receptors in engineered NK cells with or without Dex. **(D)** Volcano plot showing differentially expressed genes between wild-type (WT) NK cells and dexamethasone-treated WT (WT-Dex) cells. Genes significantly upregulated in WT-Dex and WT were colored red and blue, respectively (|log2 fold change| ≥ 0.5, adjusted *p*-value < 0.05). **(E)** Heatmap showing DEGs between WT and WT-Dex NK cells. **(F** and **G)** Bubble plots showing functional ontology terms enriched in **(F)** WT-Dex and **(G)** WT cells. Bubble size indicates the number of genes associated with each term, and color represents the functional category. Top enriched biological processes and signaling pathways were shown. **(H)** Gene set enrichment plots illustrating cytotoxicity-associated gene sets in engineered CAR-NK cells. **(I)** Genome browser tracks showing chromatin accessibility peaks at the *SPON2*, *KIT*, *TRPM8*, and *CES4A* loci. WT, unedited primary NK cells cultured without Dex; WT+Dex, unedited primary NK cells cultured with 0.5 μM Dex; Mock, NK cells subjected to electroporation with Cas9 but without sgRNA and donor DNA; KI ± Dex, TGFBR2-knockout CAR knock-in NK cells generated with or without Dex during manufacturing.

Dexamethasone is widely recognized for its immunosuppressive effects, including the suppression of NK cell function through reduced cytokine production, downregulation of activating receptors, and impairment of cytotoxic activity—ultimately dampening anti-tumor responses 54, 55. However, accumulating evidence indicates that glucocorticoids can exert context-dependent effects on immune cells, including enhancement of NK cell activity under specific activation conditions 56. To investigate this paradox, we assessed whether Dex influences the cytotoxic activity of cytokine-activated NK cells. pNK cells were stimulated with IL-12, IL-15, and IL-18, and then evaluated for cytotoxic function with or without Dex. Consistent with its canonical immunosuppressive role, Dex suppressed cytotoxicity in unstimulated NK cells. In contrast, Dex significantly enhanced cancer-killing activity and increased expression of the activation markers CD107a and CD69 in cytokine-activated NK cells (Figure 5A, B). These findings indicate that Dex enhances NK cell cytotoxicity in a cytokine-dependent manner, rather than exerting a uniform suppressive effect. In addition to improving KI efficiency during CAR-NK manufacturing, Dex also directly supported NK cell functional competence under cytokine-stimulated conditions. Notably, CAR-NK cells generated in the presence of Dex exhibited enhanced proliferative capacity and increased surface expression of activating receptors. In both electroporation-KI and AAV-KI groups, Dex-treated NK cells displayed elevated surface expression of CD16, NKp46, NKp44, and NKG2D, compared to cells generated without Dex (Figure 5C). Together, these data demonstrate that Dex augments cytotoxic potential and activation phenotypes of cytokine-activated and engineered NK cells in a context-dependent manner.

Hence, we conducted transcriptomic profiling of Dex-treated engineered NK cells to explore underlying mechanisms. RNA-seq analysis confirmed effective TGFβRII*-*disruption, showing reduced transcript levels in CAR-KI NK cells than those in WT NK (Figure S11A, B). To identify transcriptional changes driven specifically by Dex treatment, we compared gene expression profiles between WT and WT-Dex, as well as KI and KI-Dex groups. This analysis revealed a limited number of DEGs following Dex treatment, suggesting that strong IL-12/IL-15/IL-18 stimulation may override or mask subtle Dex-induced transcriptional changes (Figure 5D and Figure S11C). Nevertheless, Dex treatment selectively induced specific pathways and gene expression programs, indicating the mechanism underlying Dex-induced NK cell activation. In WT-Dex NK cells, inflammatory signaling genes—such as *CD38* and *GZMK*—were downregulated, while genes like *IGF1* and *GLUL*, which are enriched in mTOR signaling and glucose metabolism pathways, were upregulated (Figure 5E-G). To assess whether Dex-enhanced cytotoxicity is accompanied by increased inhibitory signaling or exhaustion, we next examined the expression of immune checkpoint and exhaustion-associated markers at the transcriptional level. Analysis of RNA-seq data revealed that expression of *TIGIT* and *LAG3* was reduced in Dex-treated NK cells compared with untreated controls, whereas *HAVCR2* expression remained largely unchanged. Although *PDCD1* (PD-1) transcripts were modestly increased following Dex treatment, the magnitude of this change was limited and did not coincide with a broader exhaustion-associated transcriptional program (Figure S12). Collectively, these results suggest that Dex-enhanced NK cell activation and cytotoxicity are not associated with transcriptional signatures of accelerated exhaustion or inhibitory dysfunction. To further elucidate functional implications, we performed gene set enrichment analysis (GSEA) across all groups (WT, WT-Dex, KI, and KI-Dex) based on their cytotoxicity patterns observed in the cytotoxicity assays. Interestingly, KI-Dex cells showed significant enrichment in oxidative phosphorylation (OXPHOS) and ATP synthesis pathways, a metabolic signature associated with NK cell activation 57 (Figure 5H and Figure S13). Consistent with the Dex-induced metabolic gene program, including upregulation of metabolic regulators such as IGF1 and GLUL and enrichment of OXPHOS/ATP synthesis pathways, we sought to directly validate whether these transcriptional signatures translate into functional metabolic reprogramming at the flux level. To directly validate these transcriptional predictions at the metabolic flux level, we performed Seahorse extracellular flux analysis to measure OCR and ECAR in WT, WT+Dex, KI, and KI+Dex NK cells. KI+Dex NK cells consistently exhibited the highest basal and maximal OCR, along with significantly elevated ECAR, compared with WT and KI cells, while WT+Dex cells displayed intermediate metabolic activity (Figure S14). These results demonstrate that Dex treatment enhances both mitochondrial respiration and glycolytic flux in engineered NK cells, providing direct functional evidence of metabolic reprogramming toward increased OXPHOS and ATP production. In addition, we performed qRT-PCR to validate representative candidate genes highlighted by our transcriptomic/epigenetic analyses. Dex treatment increased expression of IGF1, GLUL, and PIK3IP1 in WT+Dex compared with WT NK cells (Figure S15), supporting the directionality of the Dex-associated gene program. Finally, ATAC-seq analysis revealed that Dex increased chromatin accessibility at transcriptional start sites (TSSs) of Dex-responsive genes rather than inducing global chromatin remodeling (Figure 5D, Figure S16A-F). We further conducted a genome-wide differential accessibility analysis and identified 20 genomic regions with significantly altered accessibility in Dex-treated compared to untreated NK cells (Figure S16G-I). Notably, Dex treatment enhanced chromatin openness at promoter regions of *CXCR4* and *PIK3IP1* (Figure S16J, K). Moreover, several genes upregulated at the transcriptional level in WT-Dex cells—such as *SPON2*, *KIT*, *TRPM8*, and *CES4A*—also exhibited increased chromatin accessibility at their regulatory regions (Figure 5I and S16I). Although not all changes in chromatin accessibility reached statistical significance, the observed trends suggest a Dex-induced chromatin remodeling associated with NK cell activation and metabolism. In summary, our multi-omics analyses revealed that Dex treatment induces metabolic changes in NK cells through transcriptional regulation, promoting OXPHOS and ATP production. These effects collectively enhance the cytotoxic potential of CAR-NK cells, supporting the utility of Dex in improving their efficacy for solid tumor immunotherapy.

### One-step KO/KI CAR-NK cells suppress solid tumor growth *in vivo*

**Figure 6:**
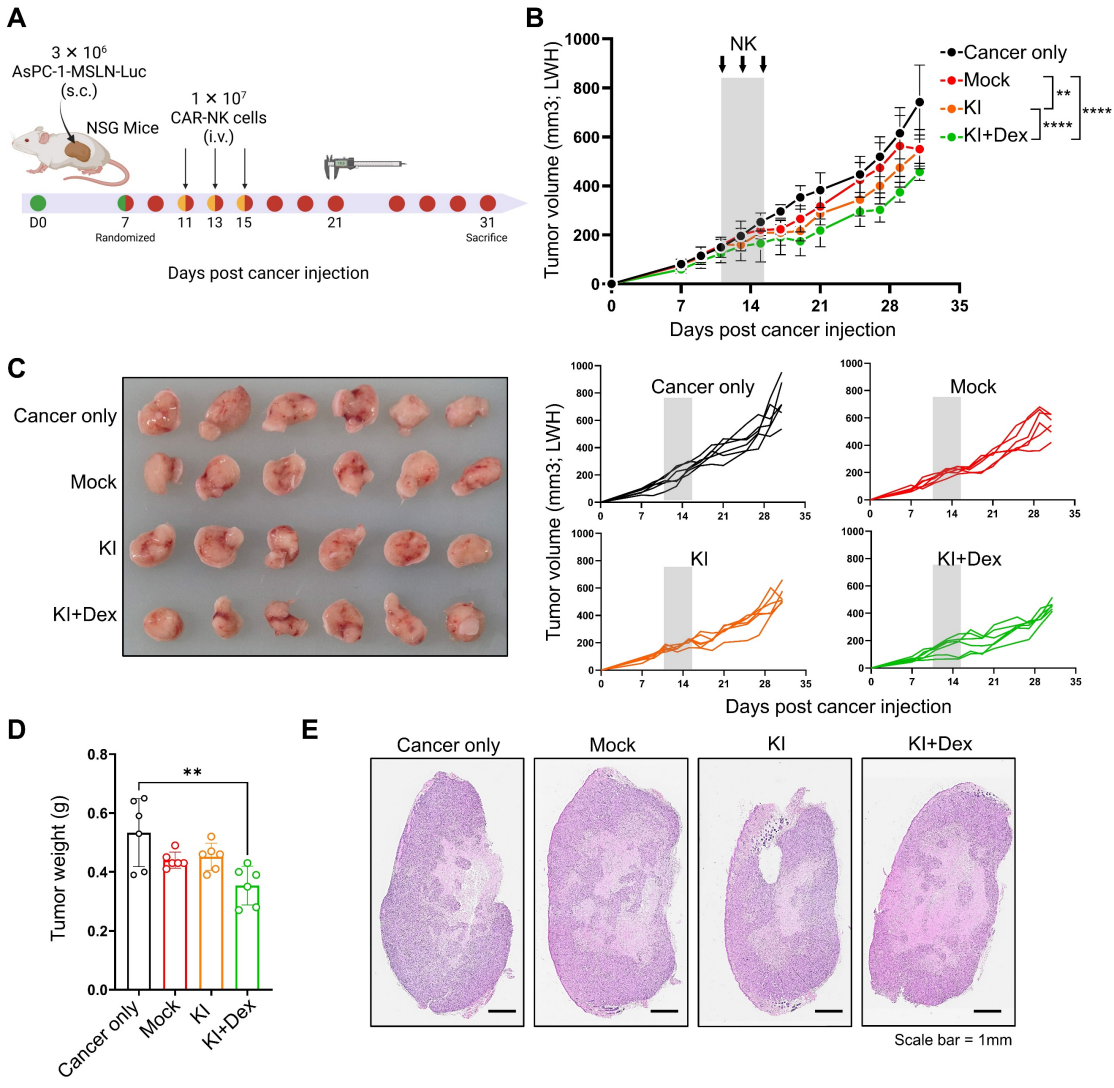
***In vivo* antitumor efficacy of one-step CAR-NK^KO/KI^ cells. (A)** Schematic overview of *in vivo* experimental design in mice bearing MSLN-overexpressing AsPC-1 tumors. **(B)** Tumor growth curves of mice. Mice were randomized into four groups: cancer only, mock NK, One-step KI NK (TGFBR2-KO, MSLN-CAR KI generated without Dex), and One-step KI+Dex NK (TGFBR2-KO, MSLN-CAR KI generated with Dex). NK cells were administered intravenously on days 11, 13, and 15 (arrows). Tumor volumes are presented as mean ± SD. Statistical significance was assessed using two-way ANOVA followed by Tukey's multiple-comparisons test; ** *p* < 0.01, **** *p* < 0.001. **(C)** Representative images of excised tumors at the experimental endpoint (left), with individual tumor growth trajectories shown for each mouse (right). **(D)** Endpoint tumor weights. Each dot represents an individual mouse. Statistical significance was determined via one-way ANOVA with post hoc multiple-comparison testing. **p < 0.01.** (E)** Representative hematoxylin and eosin (H&E)-stained tumor sections from each group at the experimental endpoint (scale bar = 1 mm).

To evaluate whether the one-step TGFBR2-targeted CAR knock-in (KO/KI) platform translates into antitumor efficacy *in vivo*, we established a subcutaneous xenograft model using MSLN-overexpressing AsPC-1 pancreatic cancer cells in immunodeficient mice (Figure 6A). Once tumors were established, animals were randomized into four groups: cancer only, mock NK (WT NK cells subjected to mock electroporation), KI NK (TGFBR2-targeted CAR KI), and KI+Dex NK (TGFBR2-targeted CAR KI generated under Dex-conditioned manufacturing). NK cells were administered intravenously on days 11, 13, and 15. Compared with cancer-only and mock NK controls, treatment with one-step-engineered CAR-NK cells generated under the KI+Dex condition significantly suppressed tumor growth, as evidenced by reduced tumor volumes over time (Figure 6B) and smaller tumors at the experimental endpoint (Figure 6C). Consistently, endpoint tumor weights were significantly lower in the KI+Dex group, whereas mock NK treatment did not significantly alter tumor burden relative to cancer-only controls (Figure 6D). Histopathological evaluation of excised tumors via hematoxylin and eosin (H&E) staining further corroborated these findings (Figure 6E). Together, these results demonstrate that CAR-NK cells produced using the virus-free, one-step KO/KI platform exhibit measurable *in vivo* antitumor activity against a solid tumor xenograft.

## Discussion

PDAC presents a formidable therapeutic challenge due to its dense fibrotic stroma, poor vascularization, and profoundly immunosuppressive TME 5, 58. A major contributor to this suppression is TGFβ1, which impairs NK cell function by reducing cytotoxicity and altering activating receptor expression 5, 6. Overcoming these TME-mediated barriers is critical to enhancing the efficacy of NK cell-based immunotherapies. Here, we developed a streamlined, electroporation-based, one-step manufacturing process for generating *TGFBR2*-KO, CAR-engineered NK cells, specifically designed to resist TGFβ1-mediated suppression in PDAC. This approach integrates CRISPR/Cas9-mediated KO of *TGFBR2* with HDR-driven KI of a CAR transgene using optimized donor DNA and electroporation conditions. Including the GRE in the donor DNA was associated with increased nuclear donor DNA abundance and improved CAR KI efficiency, particularly in the presence of Dex. Furthermore, Dex treatment during engineering markedly increased CAR expression and cancer cytotoxicity, even within the immunosuppressive context of TGFβ1 stimulation.

Our optimized “one-step CAR-NK^KO/KI^” platform offers several key advantages over conventional approaches. First, it condenses the KO and KI steps into a single electroporation, significantly simplifying the manufacturing workflow and reducing processing time—both of which are critical for clinical scalability. Second, it minimizes the extent of cell handling and manipulation, potentially preserving NK cell viability and function 59. Importantly, our strategy avoids viral vectors for CAR insertion, reducing potential safety concerns and regulatory hurdles. Nonetheless, challenges remain, including variability in HDR efficiency, integration site specificity, and off-target effects associated with CRISPR editing 60. These risks warrant further validation using whole-genome sequencing and improved sgRNA design. Our one-step KO/KI strategy is conceptually related to the seminal non-viral genome targeting approach reported by Roth and colleagues (Nature, 2018) 61, which demonstrated HDR-mediated cassette insertion in primary human T cells using Cas9 RNP complexes and donor DNA. However, direct comparison of KI efficiencies across studies should be interpreted cautiously, as HDR outcomes are influenced by multiple biological and technical variables. pNK cells differ intrinsically from T cells in DNA damage-response and repair pathways and are generally more refractory to HDR 62-65, which may limit achievable KI efficiencies. In addition, locus-dependent effects are substantial; targeting TGFBR2 exon 4 may yield different KI performance compared with loci commonly optimized in T-cell studies, such as TRAC. Differences in construct design and delivery parameters, including donor architecture and electroporation conditions, further complicate direct efficiency comparisons. Within this context, the moderate CAR KI frequencies observed in our study reflect a deliberate design choice prioritizing virus-free, locus-specific genome engineering and simultaneous functional pathway disruption. Importantly, despite these efficiencies, one-step-engineered CAR-NK cells exhibited robust functional activity *in vitro* and significant antitumor efficacy *in vivo*, supporting the translational relevance of this platform.

While a direct *in vivo* comparison between CAR-NK cells generated via this one-step platform and those produced using conventional two-step manufacturing approaches would be informative, such a comparison was beyond the scope of the current study owing to the technical and logistical challenges associated with generating additional clinical-grade viral or AAV-based CAR-NK batches for *in vivo* evaluation. Importantly, the primary objective of this work was not to benchmark manufacturing strategies head-to-head, but rather to establish the feasibility and therapeutic potential of a virus-free, one-step KO/KI platform. Consistent with this goal, CAR-NK cells generated using the one-step KO/KI strategy demonstrated robust *in vivo* antitumor activity in the subcutaneous xenograft PDAC model (Figure 6), supporting the capacity of this platform to produce functionally effective, TGFβ1-resistant CAR-NK cells for solid tumor immunotherapy.

Notably, Dex is widely known for its immunosuppressive properties 54, 66. Here, cytokine-primed NK cells (IL-2, IL15, and IL-18) exhibited enhanced cytotoxicity and CD107a expression in the presence of Dex. This paradoxical effect was preserved in engineered CAR- NK^KO/KI^ cells, which also displayed upregulation of key activating receptors, including CD16, NKp46, NKp44, and NKG2D, upon Dex treatment. To investigate the underlying mechanisms, we performed transcriptomic and epigenetic profiling. RNA-Seq confirmed TGFβRⅡ disruption in CAR-KI cells and revealed minimal gene expression changes in Dex-treated, cytokine-primed NK cells, likely due to dominant IL-12/15/18 activation masking transcriptional effects. However, comparative analysis between WT and WT-Dex NK cells identified a subset of DEGs, marked by the downregulation of inflammatory pathways and upregulation of mTOR signaling and glucose metabolism, a pattern suggestive of metabolic reprogramming 67. Among these, *CD38* and *GZMK* were significantly downregulated following Dex treatment. Inhibition of *CD38* has been reported to improve immune effector function by restraining glycolytic metabolism 68. *GZMK* has also been associated with exhaustion-like phenotypes in cytotoxic lymphocytes, and reduced *GZMK* expression aligns with a reduction in exhaustion-related features 69-71. Together, these changes are consistent with a more functional cytotoxic profile. Consistent with this, GSEA further revealed upregulation of OXPHOS and ATP synthesis pathways in Dex-treated CAR-NK^KO/KI^ cells, aligning with enhanced energy metabolism and effector function. In the resting state, a low metabolic rate is sufficient to support basal immune activity. However, upon activation with cytokines, NK cells require a high level of energy to sustain cytotoxic functions and cytokine secretion, which leads to upregulation of both glycolysis and OXPHOS to generate ATP 56, 72, 73. Dex treatment appears to facilitate this metabolic shift, enhancing effector function by promoting energy-generating pathways. In contrast, TGFβ suppresses NK cell metabolism by inhibiting mTORC1 signaling, resulting in reduced OXPHOS and glycolysis. This leads to impaired mitochondrial function, decreased ATP production, and ultimately diminished cytotoxicity and cytokine output 13, 74. Therefore, the combination of *TGFβR2* KO and Dex treatment promotes metabolic reprogramming, enabling CAR-NK cells to enhance their bioenergetic profile and effector capabilities. Supporting this, ATAC-seq identified increased chromatin accessibility at genes involved in NK migration, activation, and metabolism-including *CXCR4, PIK3IP1, SPON2, KIT, TRPM8, and CES4A*. These changes suggest that Dex modulates the NK cell chromatin accessibility landscape, potentially enhancing transcriptional readiness for cytotoxic responses. Several of these genes warrant further functional investigation. CXCR4 is essential for NK cell development, trafficking, and retention in tissues 75, while KIT receptor (CD117) enhances proliferation in response to IL-2 and stem cell 76. Although the roles of SPON2, TRPM8, and CES4A in NK cells remain less defined, they are implicated in immune cell migration, calcium signaling, and lipid metabolism, respectively 77-79. PIK3IP1, a negative regulator of PI3K-AKT signaling, may modulate the survival and metabolic tuning of NK cells under stress 80. Collectively, our findings provide a compelling case for Dex to enhance CAR-NK cell functionality through changes in chromatin accessibility and metabolic state, alongside increased genome editing efficiency. This dual benefit positions Dex as a promising addition to CAR-NK^KO/KI^ cell manufacturing protocols, particularly for targeting TGFβ-rich solid tumors like PDAC.

## Conclusions

In conclusion, our study establishes a clinically relevant, virus-free, one-step electroporation approach for producing functionally enhanced CAR-NK^KO/KI^ cells resistant to TGFβ1-mediated immunosuppression. Moreover, Dex acts as a powerful adjuvant, not only improving genome editing and CAR integration but also enhancing NK cell cytotoxicity through metabolic and chromatin remodeling. These insights provide the foundation for developing next-generation CAR-NK therapies tailored for solid tumor immunotherapy and further clinical investigation.

## Supplementary Material

Supplementary figures.

Supplementary tables.

## Data Availability

The datasets used and/or analyzed during the current study are available from the corresponding author upon reasonable request. The RNA-seq and ATAC-seq data have been deposited in the GEO database under the accession numbers GSE299464 and GSE299308, respectively.

## Abbreviations

CAR: chimeric antigen receptor
NK cells: natural killer cells
TME: tumor microenvironment
TGFβ: transforming growth factor-β
TGFβRII: TGFβ receptor II
PDAC: pancreatic ductal adenocarcinoma
ECM: extracellular matrix
CAFs: cancer-associated fibroblasts
PBMCs: peripheral blood mononuclear cells
scFv: single-chain variable fragment
T7E1: T7 endonuclease I
KO: knock-out
sgRNA: single guide RNA
RNPs: ribonucleoprotein complexes
p-SMAD2/3: phosphorylated SMAD2/3
KI: knock-in
dsDNA: double-stranded DNA
HDR: homology-directed repair
DSB: double-strand break
HA: homology arm
pEFS: EF1α short promoter
pSFFV: spleen focus-forming virus promoter
poly-A: polyadenylation tail
SV40: simian vacuolating virus 40 binding element
CREB: cyclic AMP response element-binding protein binding element
GRE: glucocorticoid response element
Dex: Dexamethasone
DEGs: differentially expressed genes
GSEA: gene set enrichment analysis
OXPHOS: oxidative phosphorylation
ATAC-seq: assay for transposase-accessible chromatin using sequencing
pNK: human primary NK
WES: Whole exome sequencing
IGV: Integrative Genimics Viewer
gDNA: genomic DNA
NSG: NOD-Prkdc^em1Baek^Il2rg^em1Baek^ mice
OCR: oxygen consumption rate
ECAR: extracellular acidification rate
GSEA: gene set enrichment analysis
TSSs: transcriptional start sites
