# Supplementary material for: One-step knock-in CAR constructs in human NK cells enable scalable, TGFβ1-resistant immunotherapy for solid tumors
Source: Theranostics. 2026 Mar 25;16(10):5609–29. doi: 10.7150/thno.127565 (PMC13080810; doi:10.7150/thno.127565)
Supplement: Supplementary file 1 — Supplementary figures. [file thnov16p5609s1.pdf]

One-step Knock-in CAR Constructs in Human NK Cells Enable Scalable, TGFβ1-resistant Immunotherapy for Solid Tumors

Su-Min Yee[1,†], Ji Hye Jeong[2,†], Daeun Kim[3,4,5,†], Sung-Bae Kang[1,6], Hanna Yoon[7], Hyein Cho[4], Daechan Park[4,8,*], Eunsung Jun[2,9,*] and Mihue Jang[1,6,10,*]

[1]*Medicinal Materials Research Center, Biomedical Research Division, Korea Institute of Science and Technology, Seoul 02792, Republic of Korea*

[2]*Department of Convergence Medicine, Asan Institute for Life Sciences, University of Ulsan College of Medicine and Asan Medical Center, Seoul 05505, Republic of Korea*

[3]*Institute of Chemical Processes, Seoul National University, Seoul 08826, Republic of Korea*

[4]*Department of Molecular Science and Technology, Ajou University, Suwon 16499, Republic of Korea*

[5]*Ajou Energy Science Research Center, Ajou University, Suwon 16499, Republic of Korea*

[6]*KHU-KIST Department of Converging Science and Technology, Kyung Hee University, Seoul 02447, Republic of Korea*

[7]*Rare & Pediatric Cancer Branch, Research Institute, National Cancer Center, Goyang 10408, Republic of Korea*

[8]*College of Advanced Bio-Convergence Engineering, Ajou University, Suwon 16499, Republic of Korea*

[9]*Division of Hepato-Biliary and Pancreatic Surgery, Department of Surgery, University of Ulsan College of Medicine, Asan Medical Center, Seoul 05505, Republic of Korea*

[10]*Division of Bio-Medical Science and Technology, KIST School, University of Science and Technology (UST), Seoul 02792, Republic of Korea*

† Su-Min Yee, Ji Hye Jeong, and Daeun Kim contributed equally to this work.

*Correspondence

Mihue Jang, *email: mihue@kist.re.kr, Tel: +82-2-958-6618, fax: +82-2-958-5909*

Eunsung Jun, *email: eunsungjun@amc.seoul.kr, Tel: +82-2-3010-1696; fax: +82-2-2045-4146*

Daechan Park, *email: dpark@ajou.ac.kr, Tel: +82-31-219-2514*

Supplementary Figures

A

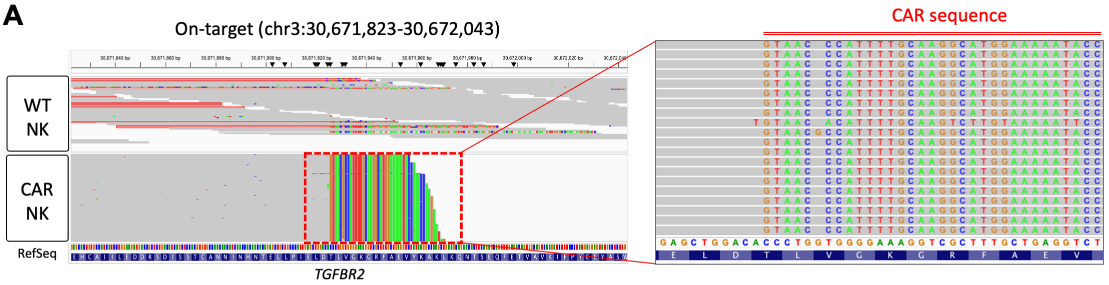

B

sgRNA	Index	Off-target DNA	Chromosome	Coordinates	#Mismatches	Gene / Region
GCCCATTGAGCTGGACACCCNGG	①	GGTCATGGAGCTGGACACCCAGG	chr8	69,324,287	3	- / intergenic
	②	GGCCATGAAGCTGGACACCCGGG	chr5	1,618,670	3	PDCD6P1 / intron
	③	GCCCTTTGAGCTGGTCATCCAGG	chr7	49,298,804	3	- / intergenic
	④	GGCCATTGAGCTGCACCCCCAGG	chr2	12,649,657	3	MIR3681HG / intron
	⑤	GCTCCTTGAGCTGGAAACCCTGG	chr2	239,944,752	3	NDUFA10 / intron
	⑥	CCCCAGTGAGATGGACACCCTGG	chr17	77,796,928	3	- / intergenic
	⑦	GGCCATGGAGCTGGAGACCCGGG	chr3	196,000,543	3	ENSG00000286168 / intron

C

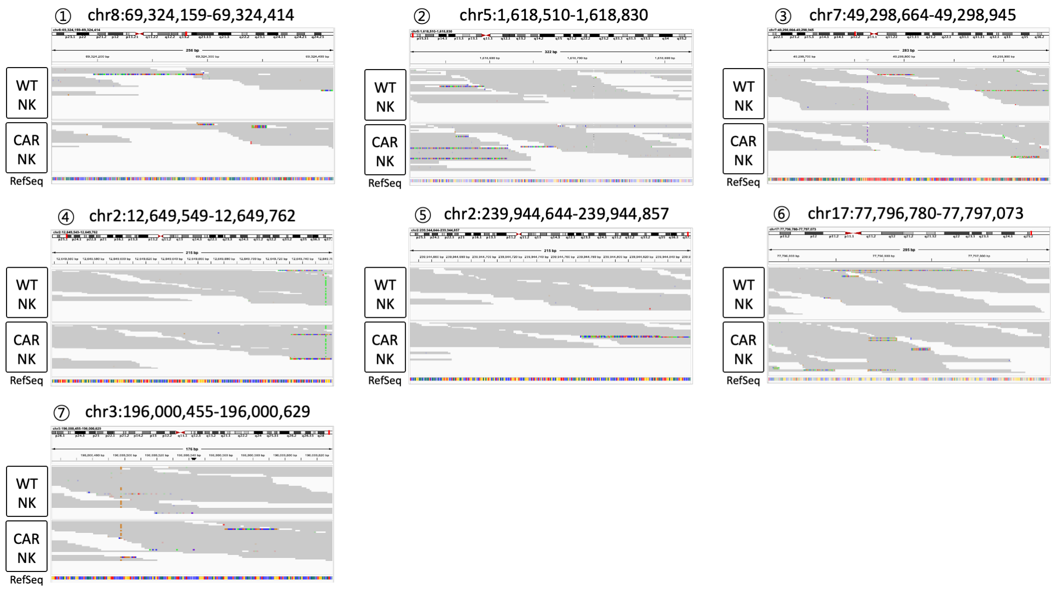

Figure S1. Validation of on-target chimeric antigen receptor (CAR) integration and off-target analysis using whole exome sequencing.

(A) Integrative Genomics Viewer (IGV) visualization of sequencing reads at the on-target site (*TGFBR2* exon 4) in WT and CAR-NK cells, showing chimeric reads comprising genomic *TGFBR2* and the CAR sequence. **(B)** Predicted off-target sites identified by Cas-OFFinder with three mismatches relative to the single guide RNA (sgRNA) target sequence. **(C)** IGV snapshots of the predicted off-target loci showing no detectable chimeric reads containing the CAR sequence.

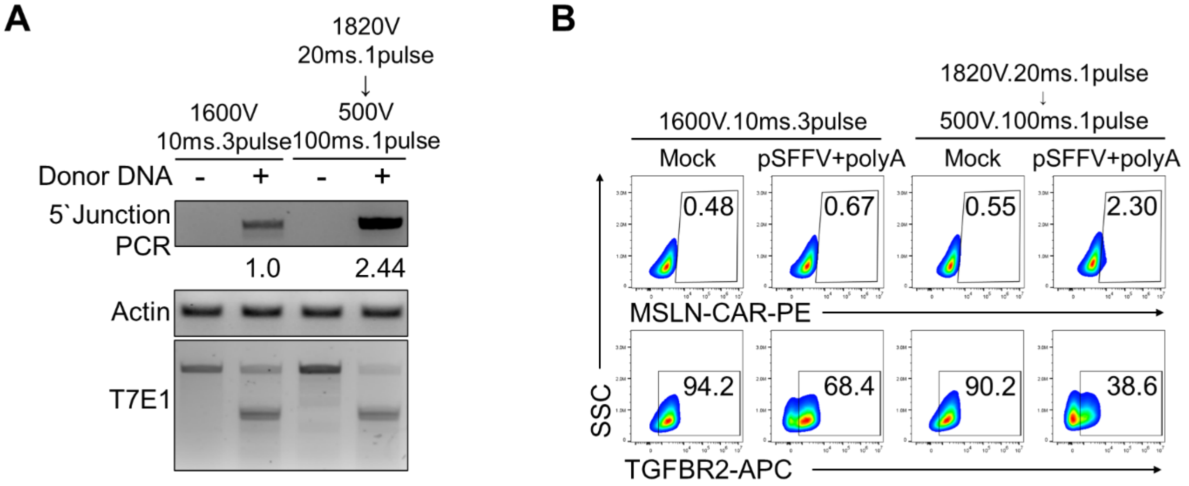

Figure S2. Validation of optimized CAR knock-in (KI) using 5' junction PCR, T7E1 assay, and flow cytometry.

(A) 5' Junction PCR and T7E1 assay results confirming CAR KI and *TGFBR2* knock-out (KO) under two different electroporation conditions. (**B)** Flow cytometry analysis of CAR and TGFβRII expression levels in NK cells engineered under electroporation.

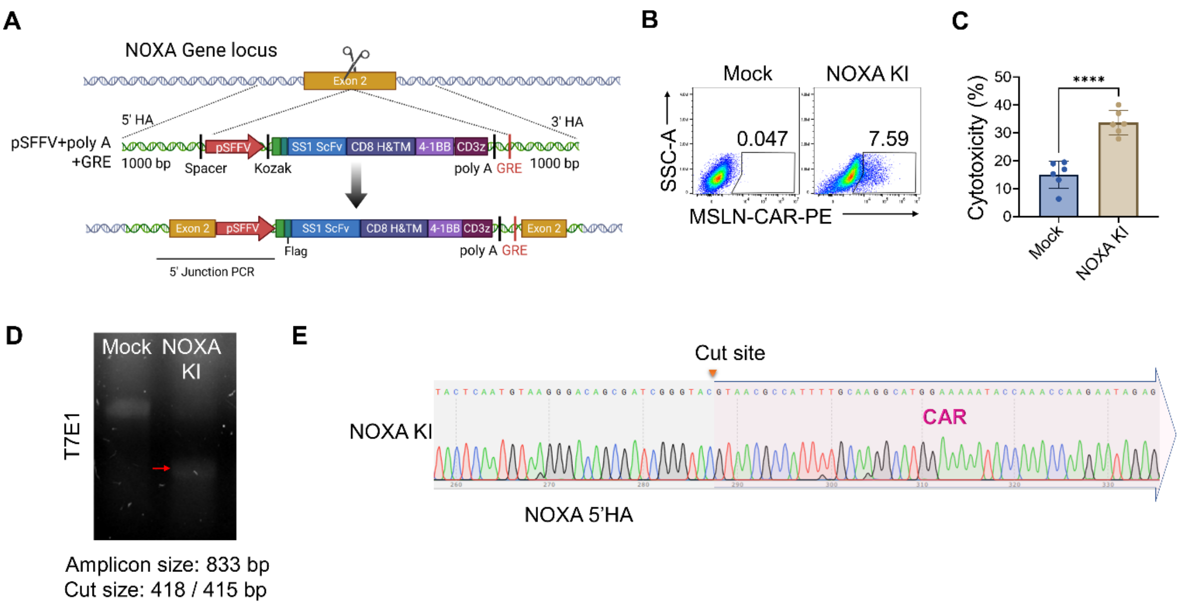

Figure S3. Validation of CAR KI at the NOXA locus in primary NK (pNK) cells.

(**A**) Schematic diagram of HDR knock-in for NOXA gene locus. (**B**) Flow cytometry analysis of mesothelin CAR (MSLN-CAR) expression in pNK cells under mock control or NOXA KI conditions. Percentages indicate CAR$^+$ cells within live NK cells. (**C**) Cytotoxicity against MSLN-overexpressing AsPC-1 target cells measured at E:T = 1:1 after 24 h co-culture. (**D**) T7E1 assay for confirming knock-out of NOXA locus in KI conditions (expected band size: 833 bp; internal fragments 418/415 bp as indicated). (**E**) Sanger sequencing chromatograms verifying correct KI junction sequence in NOXA KI conditions. *p*-values were calculated using Student's *t*-test. Data are shown as mean ± SD. ****p <0.0001.

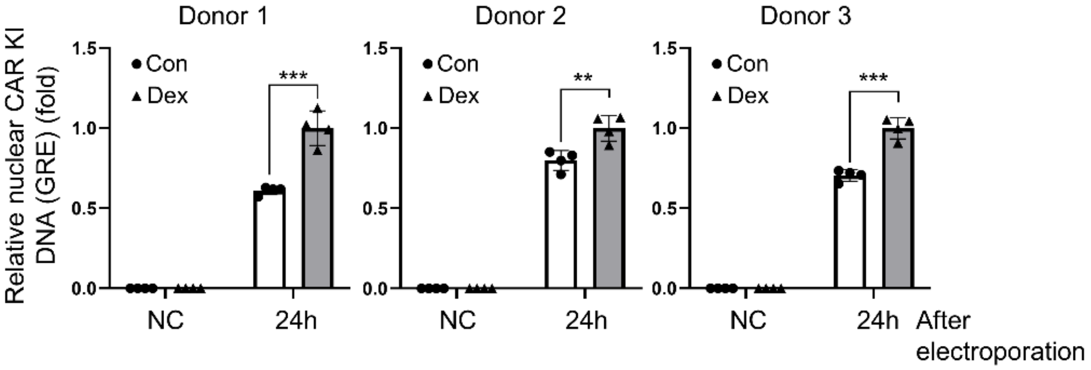

Figure S4. Dexamethasone (Dex) increases nuclear accumulation of donor DNA after electroporation.

pNK cells were electroporated with Cas9 RNPs and a glucocorticoid response element (GRE)-containing donor dsDNA, followed by culture ± Dex (0.5 μM). At 24 h post-electroporation, nuclei were isolated using a nuclear extraction kit and genomic DNA was purified from the nuclear fraction. Nuclear donor DNA abundance was quantified by qPCR. *p*-values were calculated using a two-tailed unpaired Student's *t*-test between 24 h con vs. 24 h Dex. Data are shown as mean ± SD from n = 4 biological replicate with 3 independent NK donors. $^{**}p < 0.01$ and $^{***}p < 0.001$.

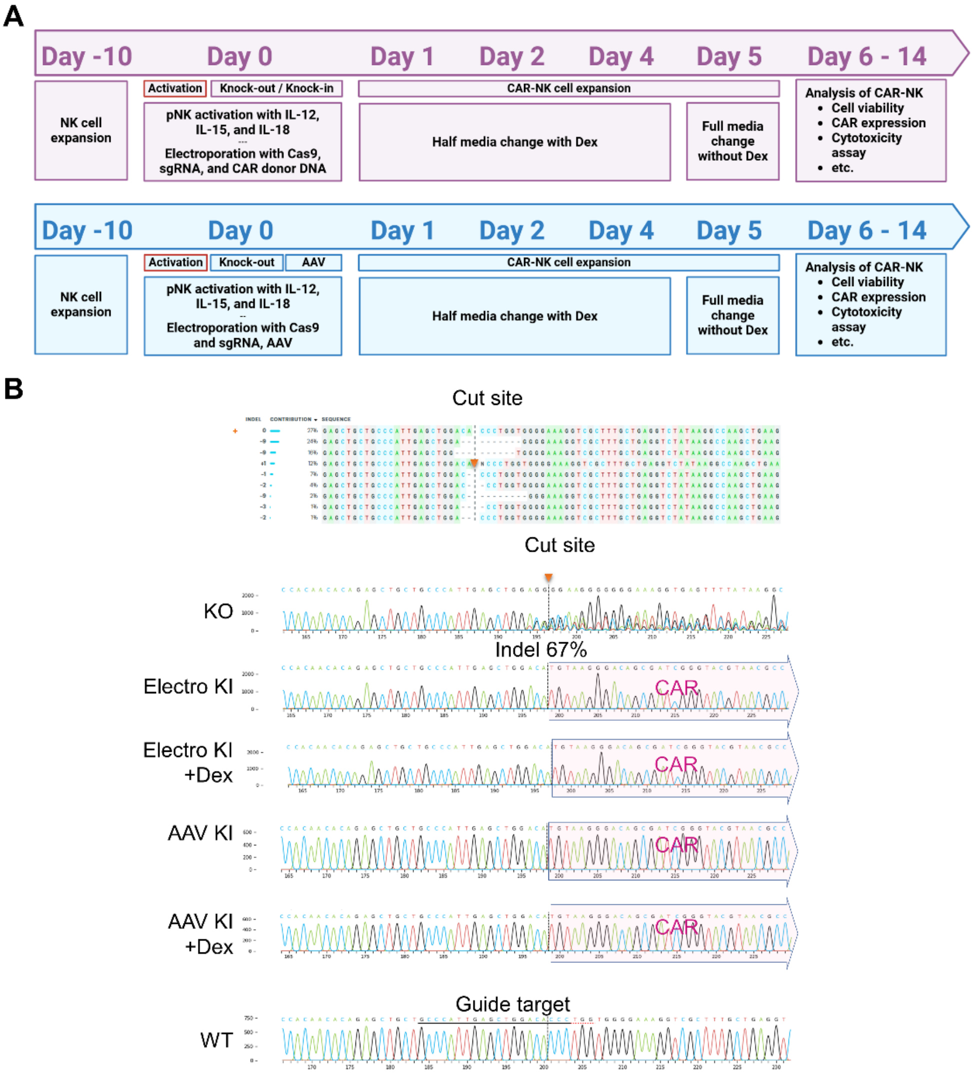

Figure S5. Experimental timeline and validation of gene editing and CAR expression in engineered NK cells.

(A) Schematic overview of two experimental timelines for CAR-NK$^{KO/KI}$ cell generation: CAR KI using Cas9, sgRNA, and donor DNA (top), and CAR introduction using Cas9, sgRNA, and adeno-associated virus (AAV) (bottom). Both protocols involved initial NK cell activation with IL-12, IL-15, and IL-18, followed by Dex treatment during CAR-NK cell expansion. **(B)** Representative indel sequences and mutation frequencies at the sgRNA-targeted *TGFBR2* exon 4 locus following spCas9 RNPs and donor DNA treatment, along with Sanger sequencing chromatograms confirming CAR KI at the target site.

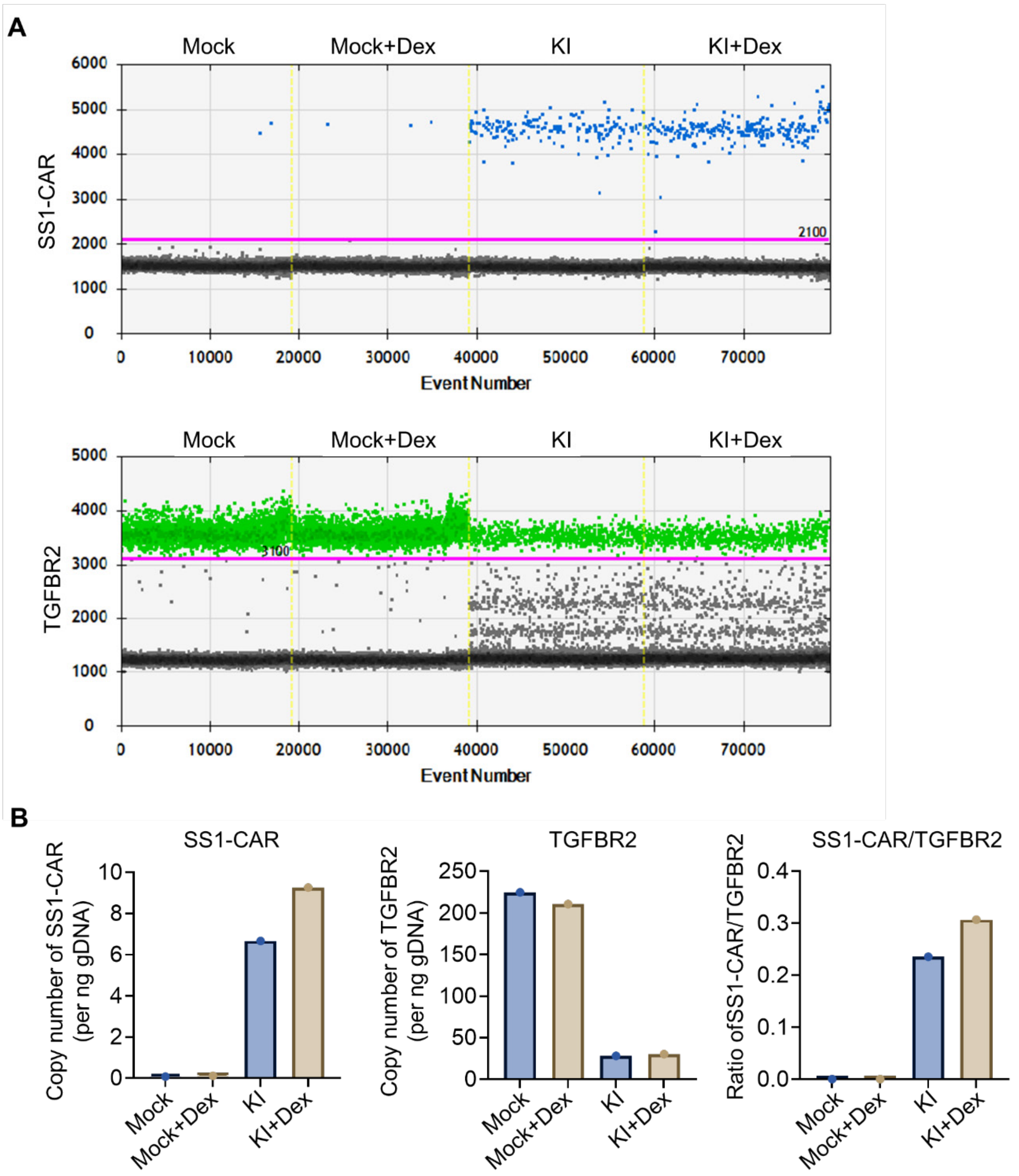

Figure S6. Droplet Digital PCR (ddPCR) confirms increased genomic CAR donor abundance in KI+Dex NK cells.

(**A**) Representative droplet amplitude plots from duplex ddPCR showing detection of the CAR (SS1) target (Channel 1; FAM) and the genomic reference TGFBR2 (Channel 2; HEX). Thresholds were set at 2100 (Ch1) and 3100 (Ch2) to discriminate positive and negative droplets. (**B**) Absolute copy numbers (copies/ng gDNA) and the normalized CAR-to-TGFBR2

ratio (SS1/TGFBR2) were calculated using QuantaSoft (Poisson correction) from genomic DNA isolated from Mock, Mock-Dex, one-step KI and one-step KI+Dex NK products.

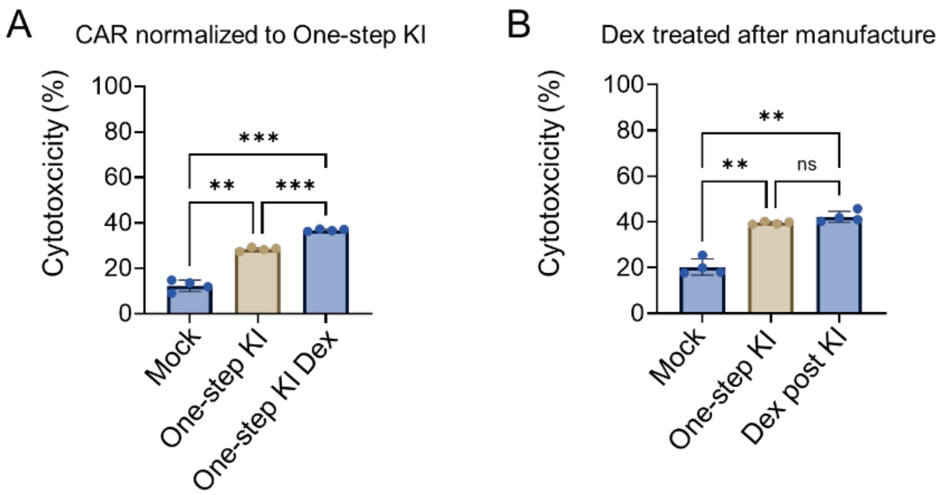

Figure S7. Dex-associated enhancement of cytotoxicity is not explained by CAR^{+} frequency and depends on manufacturing-phase exposure.

(**A**) CAR-normalized cytotoxicity analysis. Cytotoxicity against AsPC-1-MSLN-Luc target cells was analyzed by normalizing effector CAR^{+} percentage. (**B**) Post-KI Dex exposure. After completion of the KI manufacturing process, Dex (0.5 μM) was added during post-edit culture to assess whether Dex enhances cytotoxicity independent of knock-in efficiency. Cytotoxicity was assessed at E:T = 1:1 after 24 h co-culture. *p*-values were calculated using one-way ANOVA followed via Tukey's multiple-comparisons test. Data are shown as mean ± SD from n = 4 biological replicates. $^{**}p < 0.01$ and $^{***}p < 0.001$; ns, not significant.

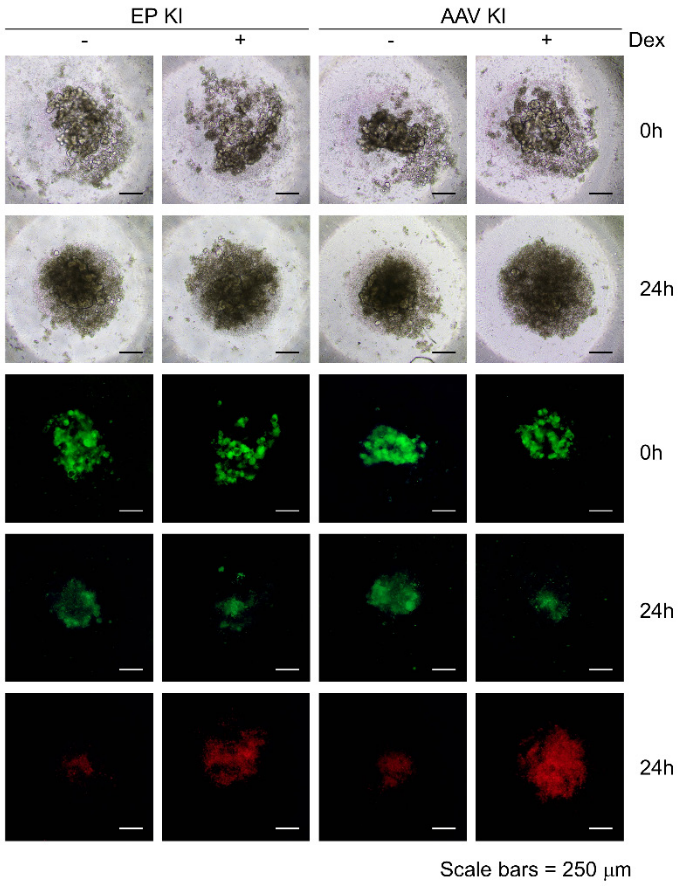

Figure S8. CAR-NK$^{KO/KI}$ -mediated tumor spheroid killing assay.

Brightfield images of tumor spheroids co-cultured with CAR-NK cells, alongside correspondi ng fluorescence microscopy images: GFP-expressing patient-derived cancer organoids (green) indicate viable organoid cells, whereas red fluorescence marks Caspase 3/7 activation, indicat ing cell death.

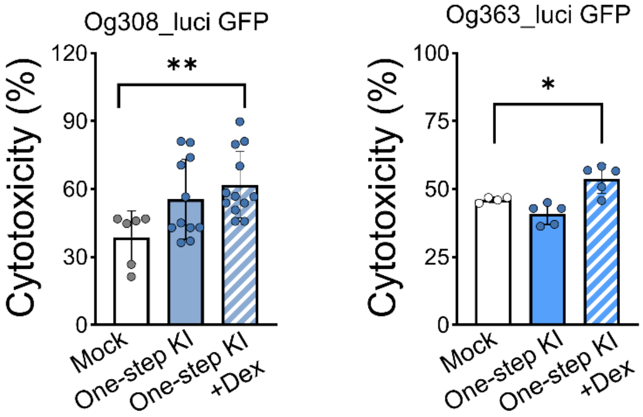

Figure S9. Cytotoxic activity of engineered CAR-NK cells against two independent patient-derived pancreatic cancer organoids.

Organoids expressing GFP and luciferase were co-cultured with electroporated Mock, one-step CAR-KI, or one-step CAR-KI + Dex NK cells, and cytotoxicity was quantified based on GFP reduction. Each organoid line was tested using NK cells from a different donor. Each dot represents an independent biological replicate. Data are shown as mean ± SD. Statistical significance is indicated (*$p < 0.05$, **$p < 0.01$).

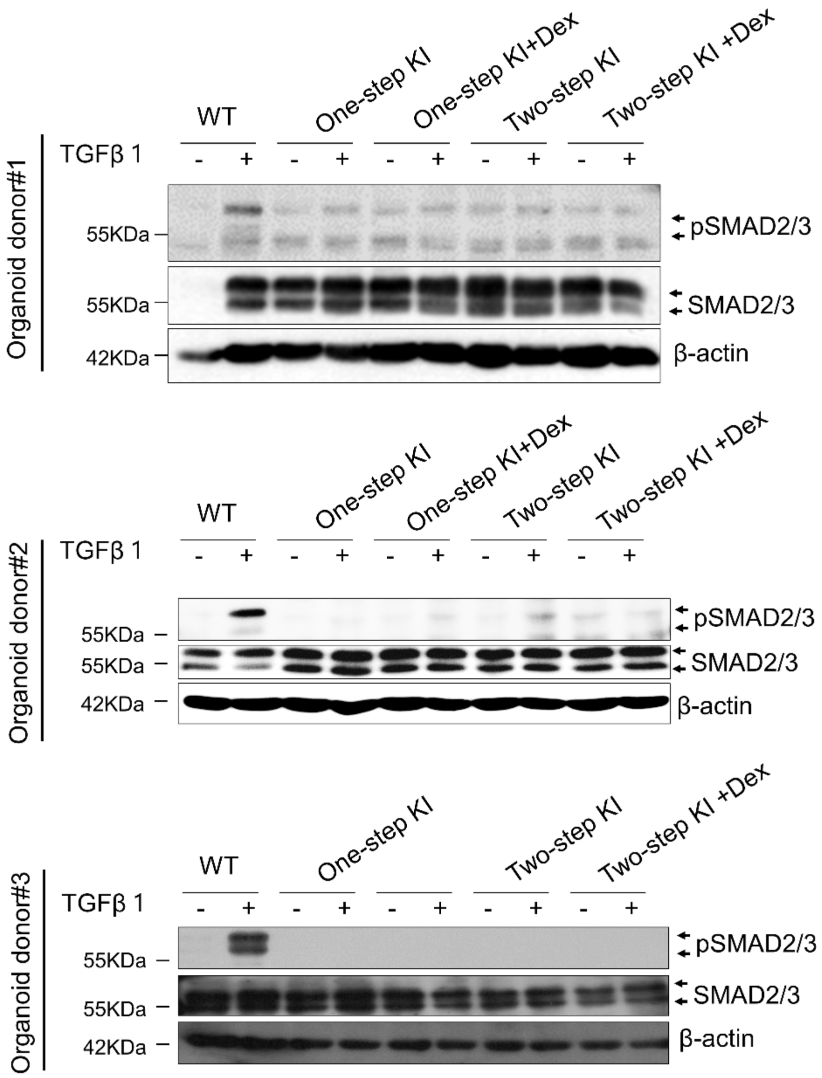

Figure S10. Functional suppression of TGFβ1 signaling in TGFBR2-KO CAR-NK cells.

Immunoblot analysis of phosphorylated SMAD2/3 (pSMAD2/3) and total SMAD2/3 in pancreatic cancer organoid co-culture systems derived from two independent donors. WT NK cells, one-step–engineered CAR-NK cells, and two-step–engineered CAR-NK cells were analyzed following stimulation with TGFβ1, as indicated. Where shown, CAR-NK cells were generated with or without Dex during the manufacturing process. β-actin served as a loading control.

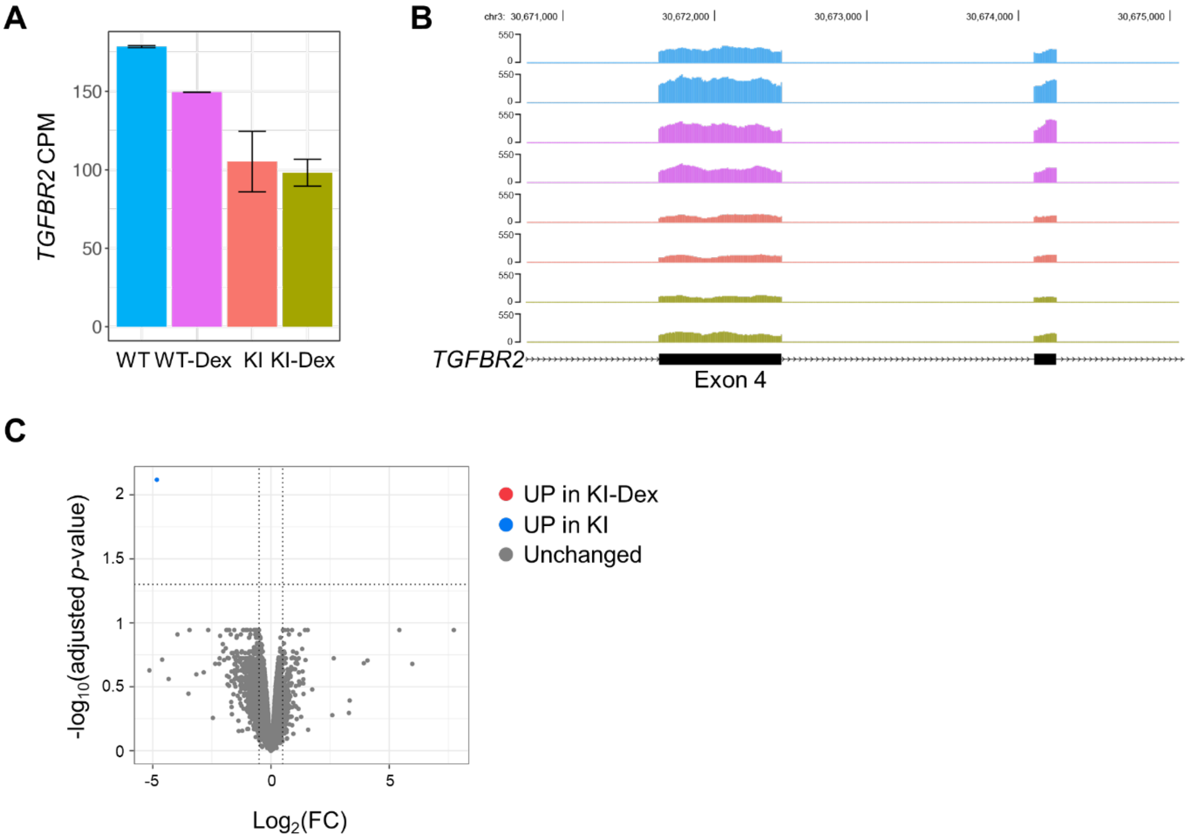

Figure S11. *TGFBR2* expression and differential gene expression in CAR-NK$^{KO/KI}$ cells with Dex treatment.

(A) Bar graph of mean normalized *TGFBR2* expression levels across all samples, with error bars indicating standard error of the mean. CPM indicates counts per million. (**B)** Genome browser visualization showing read coverage at the *TGFBR2* exon4 locus in CAR-NK$^{KO/KI}$ cells. (**C)** Volcano plot of differentially expressed genes (DEGs) between KI and KI-Dex groups. Genes significantly upregulated in KI-Dex and KI were colored red and blue, respectively ($|\log2$ fold change$| \geq 0.5$, adjusted p-value < 0.05).

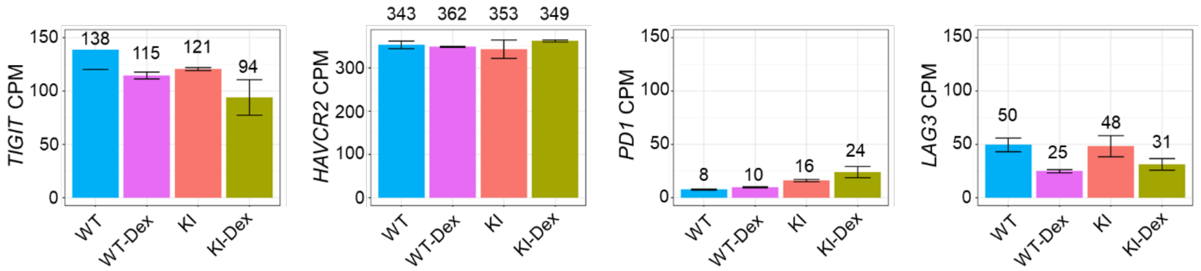

Figure S12. RNA-seq–based analysis of inhibitory and exhaustion-associated markers (*TIGIT*, *HAVCR2*, *PDCD1, LAG3*) in WT, WT+Dex, KI, and KI+Dex NK cells.

Dex treatment reduced *TIGIT* and *LAG3* expression, showed comparable *HAVCR2* levels, and induced a modest increase in *PDCD1*, without evidence of a global exhaustion-associated transcriptional program. CPM indicates counts per million.

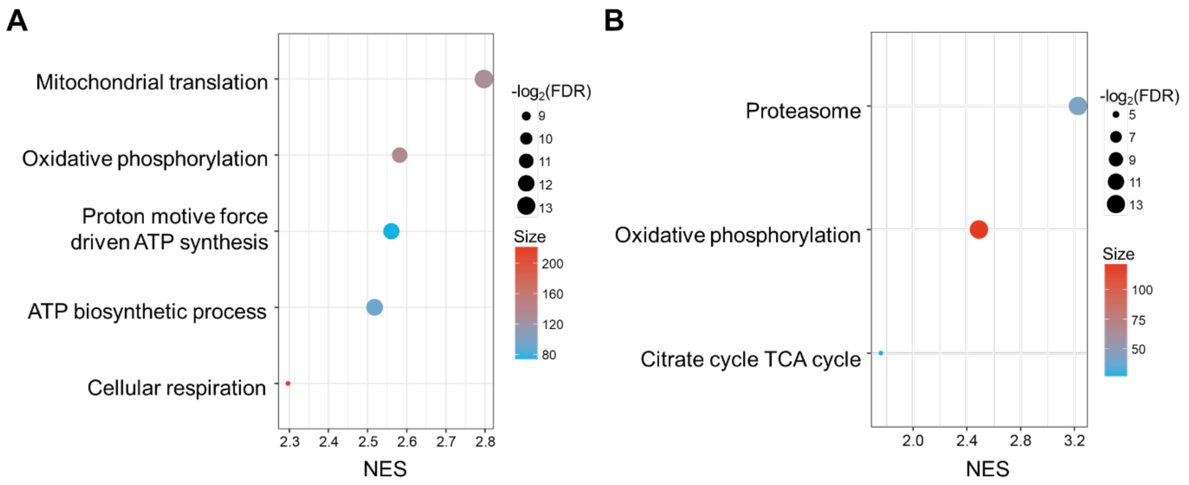

Figure S13. Functional enrichment and pathway analysis of Dex-induced transcriptomic alterations.

Gene Ontology (GO) **(A)** and Kyoto Encyclopedia of Genes and Genomes (KEGG) **(B)** pathway enrichment analysis of genes related to NK cell cytotoxicity. Bubble size represents the number of genes associated with each term, and color intensity reflects statistical significance. Top enriched biological processes and signaling pathways were shown.

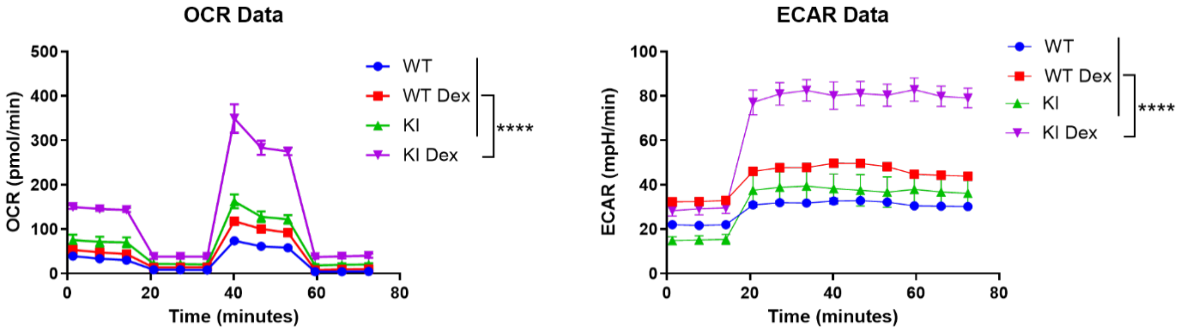

Figure S14. Seahorse extracellular flux analysis confirms Dex-associated metabolic reprogramming in engineered NK cells.

Oxygen consumption rate (OCR; left) and extracellular acidification rate (ECAR; right) were measured using Seahorse extracellular flux analysis in WT, WT+Dex, KI, and KI+Dex NK cells. For OCR and ECAR, measurements were obtained using the Seahorse Mito Stress Test, with sequential injections of oligomycin, FCCP, and rotenone/antimycin A. *p*-values were calculated using two-way ANOVA with Dunnett's multiple-comparisons test. Data are shown as mean ± SD with four biological replicates. ****$p < 0.0001$.

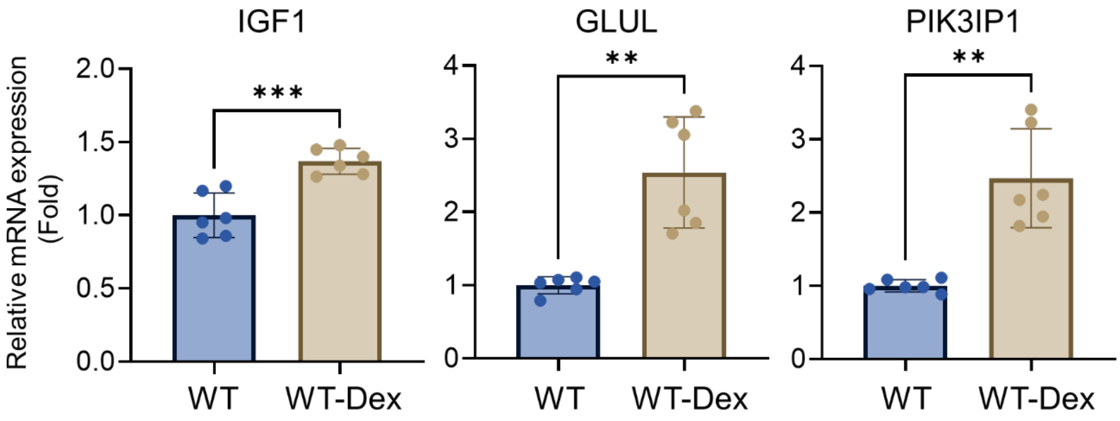

Figure S15. qRT–PCR validation of Dex-associated candidate genes in NK cells.

qRT–PCR was performed on RNA from WT and WT+Dex NK cells to quantify expression of candidate genes (IGF1, GLUL, and PIK3IP1). Expression was normalized to GAPDH and plotted as fold-change relative to WT (ΔΔCt). The graph shown as mean ± SD, n = 6. Statistical significance was determined by Student's *t* test, $^{**}p < 0.01$ and $^{***}p < 0.001$

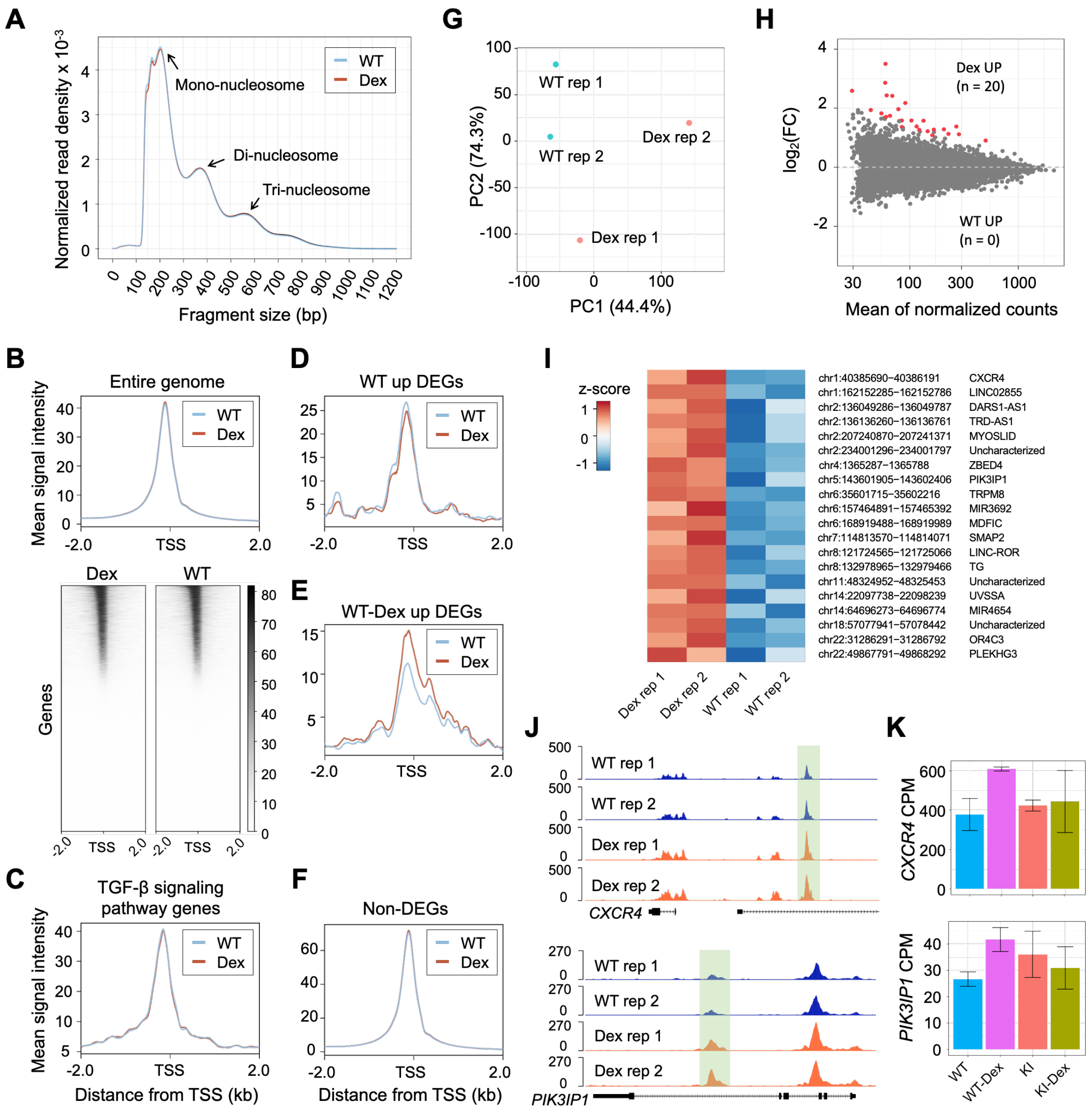

Figure S16. Chromatin accessibility dynamics in NK cells upon Dex treatment.

(A) ATAC-seq fragment length distribution of WT and Dex samples. Histograms show periodicity patterns of nucleosomes. **(B)** Average profiles and heatmaps of chromatin accessibility at transcriptional start sties (TSSs) across the genome. **(C-F)** Average profiles of chromatin accessibility at TSSs of TGF-β signaling pathway genes **(C)**, WT upregulated genes **(D)**, WT-Dex upregulated genes **(E)**, and non-DEGs **(F)** based on the comparison between WT and WT-Dex shown in Fig. 5D**. (G)** Principal component analysis (PCA) of chromatin accessibility profiles derived from ATAC-seq data. **(H)** MA plot of differentially accessible

chromatin regions between WT and Dex-treated NK cells. Regions significantly upregulated in Dex were colored red (log2 fold change| ≥ 0.5, adjusted *p*-value < 0.05). **(I)** Heatmap of differentially accessible regions annotated with nearest genes to each peak. **(J)** Genome browser tracks of representative loci show ATAC-seq peak intensities. Green-shaded regions indicate differential accessibility upon Dex treatment. **(K)** Bar plots of transcriptional levels for selected loci corresponding to differentially accessible regions. CPM indicates counts per million.

Raw images of western blots and DNA gel electrophoresis

[Figure 1E]

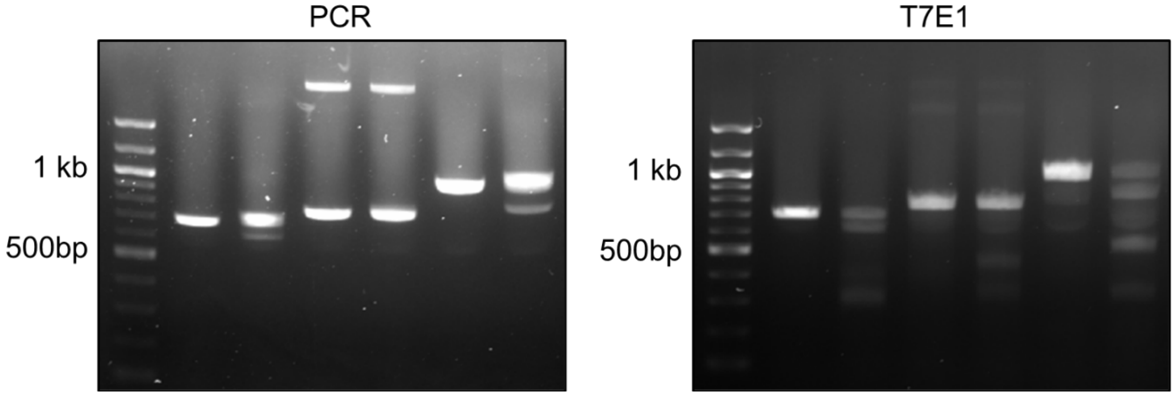

[Figure 1F]

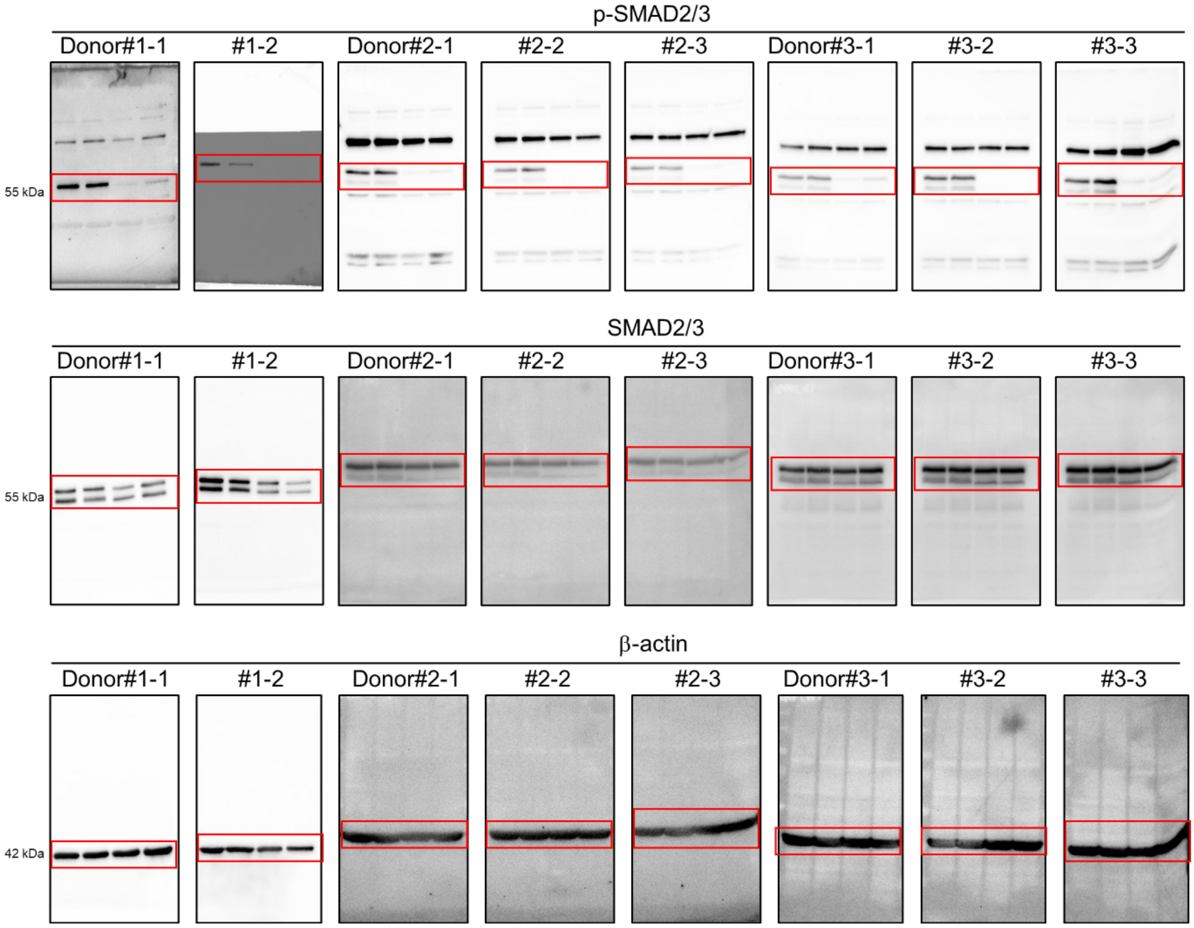
p-SMAD2/3
Donor#1-1
#1-2
Donor#2-1
#2-2
#2-3
Donor#3-1
#3-2
#3-3
55 kDa
SMAD2/3
Donor#1-1
#1-2
Donor#2-1
#2-2
#2-3
Donor#3-1
#3-2
#3-3
55 kDa
β-actin
Donor#1-1
#1-2
Donor#2-1
#2-2
#2-3
Donor#3-1
#3-2
#3-3
42 kDa

[Figure 2B]

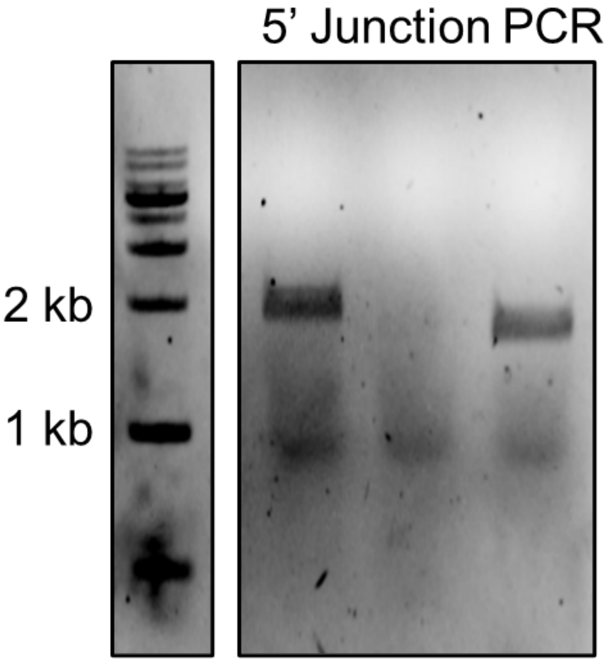

[Figure 2D]

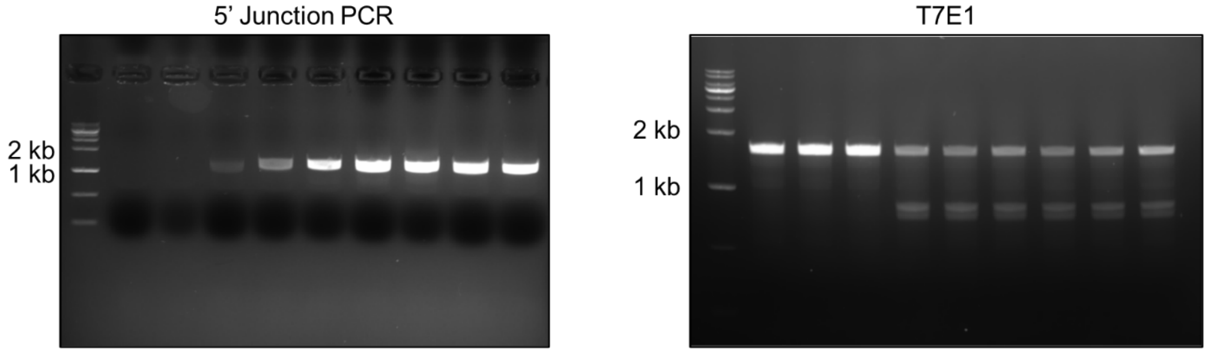

[Figure 3B]

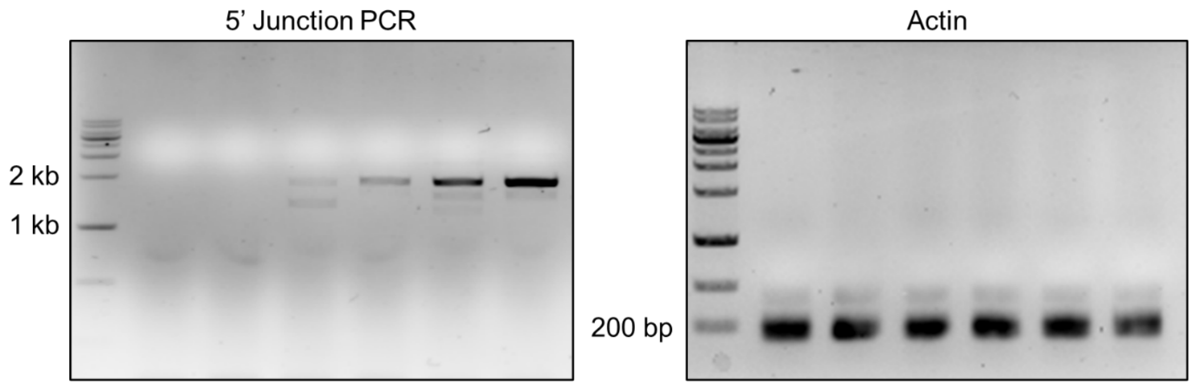

[Figure 4C]

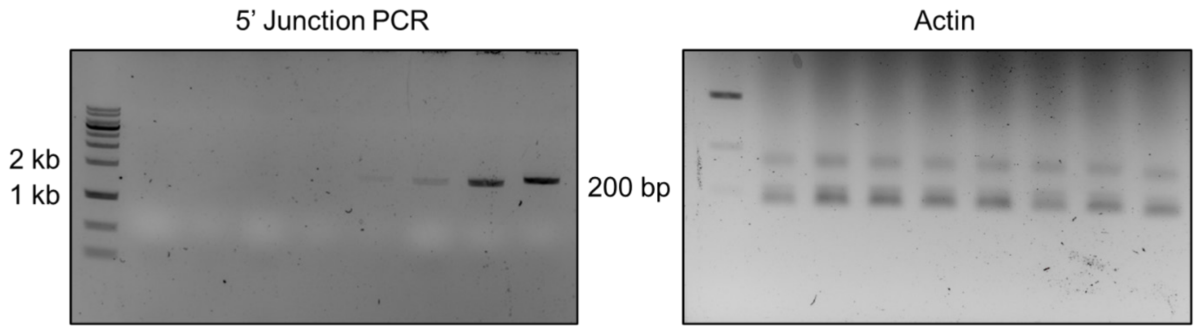

[Figure S2A]

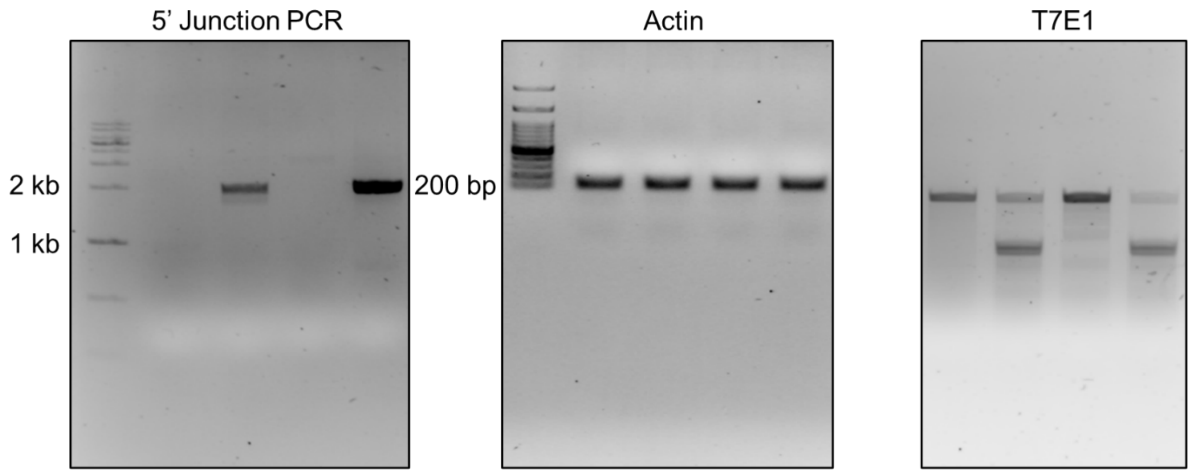

[Figure S3D]

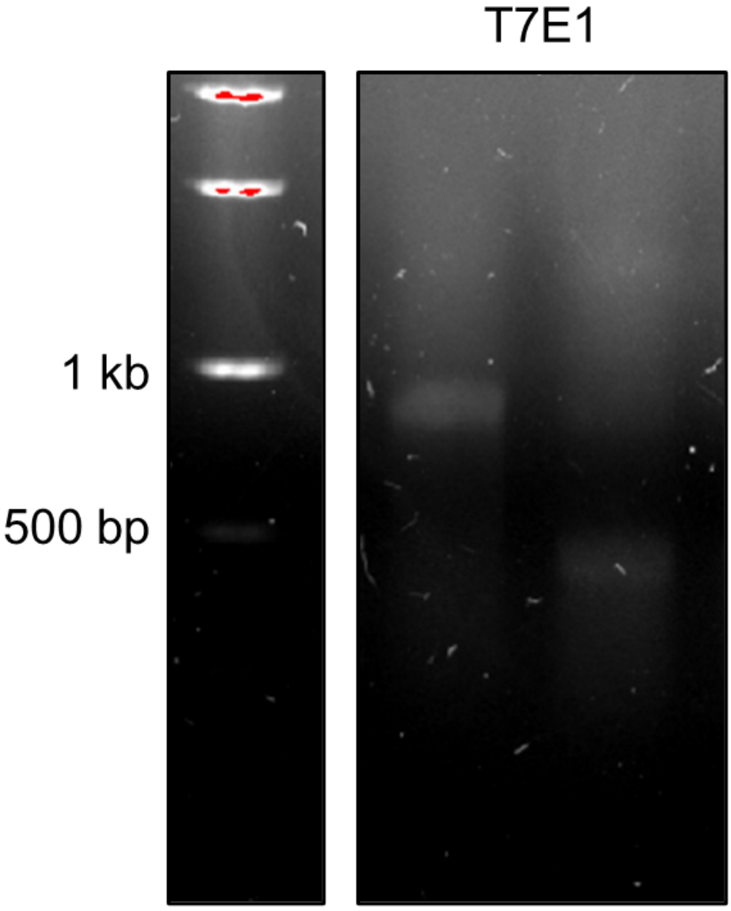

[Figure S10]

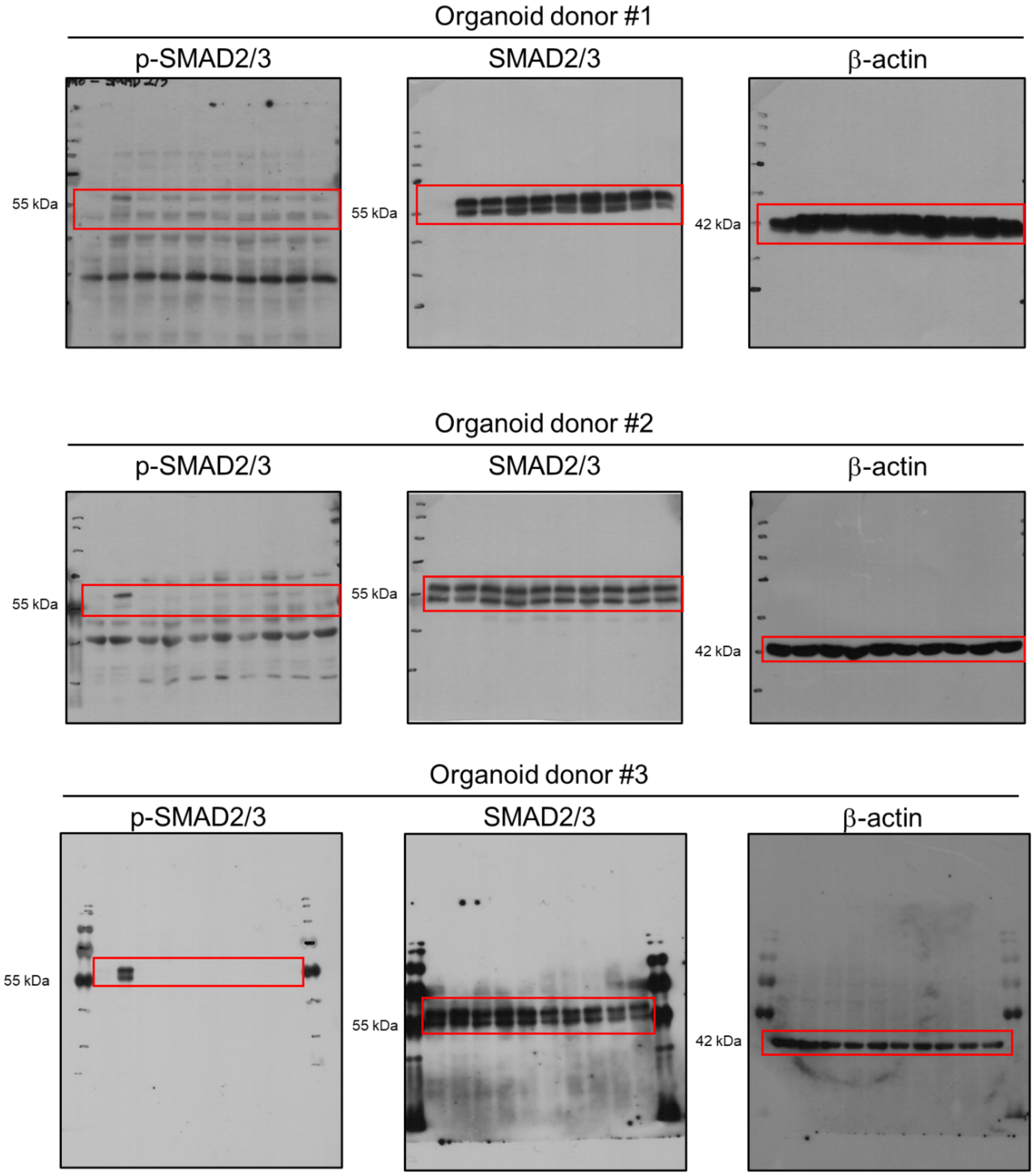
Organoid donor #1
p-SMAD2/3
SMAD2/3
β-actin
55 kDa
55 kDa
42 kDa
Organoid donor #2
p-SMAD2/3
SMAD2/3
β-actin
55 kDa
55 kDa
42 kDa
Organoid donor #3
p-SMAD2/3
SMAD2/3
β-actin
55 kDa
55 kDa
42 kDa
